# Genome-Wide Analysis Reveals PADI4 Cooperates with Elk-1 to Activate *c-Fos* Expression in Breast Cancer Cells

**DOI:** 10.1371/journal.pgen.1002112

**Published:** 2011-06-02

**Authors:** Xuesen Zhang, Matthew J. Gamble, Sonja Stadler, Brian D. Cherrington, Corey P. Causey, Paul R. Thompson, Mark S. Roberson, W. Lee Kraus, Scott A. Coonrod

**Affiliations:** 1 Baker Institute for Animal Health, College of Veterinary Medicine, Cornell University, Ithaca, New York, United States of America; 2 Department of Molecular Biology and Genetics, Cornell University, Ithaca, New York, United States of America; 3 Laboratory of Chromatin Biology, Rockefeller University, New York, New York, United States of America; 4 Department of Chemistry and Biochemistry, University of South Carolina, Columbia, South Carolina, United States of America; 5 Department of Biomedical Sciences, College of Veterinary Medicine, Cornell University, Ithaca, New York, United States of America; Max-Planck-Institute of Immunobiology, Germany

## Abstract

Peptidylarginine deiminase IV (PADI4) catalyzes the conversion of positively charged arginine and methylarginine residues to neutrally charged citrulline, and this activity has been linked to the repression of a limited number of target genes. To broaden our knowledge of the regulatory potential of PADI4, we utilized chromatin immunoprecipitation coupled with promoter tiling array (ChIP-chip) analysis to more comprehensively investigate the range of PADI4 target genes across the genome in MCF-7 breast cancer cells. Results showed that PADI4 is enriched in gene promoter regions near transcription start sites (TSSs); and, surprisingly, this pattern of binding is primarily associated with actively transcribed genes. Computational analysis found potential binding sites for Elk-1, a member of the ETS oncogene family, to be highly enriched around PADI4 binding sites; and coimmunoprecipitation analysis then confirmed that Elk-1 physically associates with PADI4. To better understand how PADI4 may facilitate gene transactivation, we then show that PADI4 interacts with Elk-1 at the *c-Fos* promoter and that, following Epidermal Growth Factor (EGF) stimulation, PADI4 catalytic activity facilitates Elk-1 phosphorylation, histone H4 acetylation, and *c-Fos* transcriptional activation. These results define a novel role for PADI4 as a transcription factor co-activator.

## Author Summary

Peptidylarginine deiminase 4 (PADI4) converts positively charged arginine and methylarginine residues on histones to the neutrally charged non-standard amino acid citrulline. We and others have previously shown that citrullination of a small subset of gene promoters, such as the estrogen receptor target, *TFF1*, appears to downregulate gene expression. In this study, we looked across the human genome using ChIP-chip to better define the full repertoire of genes that are regulated by PADI4 in breast cancer cells. Surprisingly, we found that PADI4 appears to primarily be involved in gene activation, as opposed to gene repression. Further, we found that PADI4 is likely recruited as a co-activator to these target genes by a range of well-defined transcription factors, such as Elk-1. With respect to how PADI4 activates gene transcription, we show that PADI4 directly interacts with Elk-1 at its well-defined target, the *c-Fos* oncogene. Additionally, we found that, following stimulation with epidermal growth factor, PADI4 appears to directly target Elk-1 for citrullination, which in turn leads to increased histone acetylation and gene transcription. These novel genome-wide and gene-specific findings suggest that PADI4 plays a much broader role in gene activation than previously thought.

## Introduction

The mitogen activated protein kinase/extracellular signal-related kinase (MAPK/ERK) pathway couples extracellular signals with a range of intracellular responses including cell growth, proliferation, and differentiation. An important target of MAPK/ERK activity is Elk-1 (Ets-like protein-1), a member of the ternary complex factors family of ETS domain transcription factors [1]. The ternary complex factors form a complex with serum response factor at the serum response element (SRE) and this *cis*-element is required for regulation of many immediate-early genes by stimulation with growth factors, such as EGF [2]–[6]. Elk-1 is activated by MAPK/ERK via phosphorylation of serine residues 383 and 389 within the transactivation domain [7], [8], leading to an enhanced association between Elk-1 and the histone actyltransferase (HAT), p300 [9]. The activated Elk-1-p300 complex then induces a strong p300-mediated acetyltransferase activity on target genes leading to enhanced histone acetylation, chromatin remodelling, and gene activation [8]–[14].

The discovery that PADI4 is a nuclear enzyme which converts histone arginine residues to citrulline [15] provided the first evidence that PADI4 may play a role in gene regulation. This prediction was validated by the findings that PADI4 mediated citrullination of histone arginine residues (both unmodified and monomethylated) at the hormone dependent *TFF1* promoter [16]–[18], and at the apoptosis related gene promoters, *p21* and *OKL38* [19], [20], represses gene transcription. However, due to the limited number of target genes identified to date, our understanding of the gene regulatory potential of PADI4 remains far from complete. Therefore, to more fully elucidate the repertoire of PADI4 target genes, in this study we first carried out ChIP-chip analysis using a PADI4-specific antibody in MCF-7 cells. Surprisingly, in addition to the previously described repressive role for PADI4 in transcription, we found that PADI4 is also associated with a large set of actively transcribed genes and appears to function as a transcriptional coactivator of at least a subset of these genes. Computational analysis then found that our PADI4 ChIP-chip data set is strongly correlated with published data sets from activating, but not repressive, markers and that several common transcription factor binding sites are enriched around PADI4 peaks, with the V$ELK1_02 motif being the most significant element. We then demonstrate that PADI4 interacts with Elk-1 and that citrullination of Elk-1 by enzymatically active PADI4 facilitates ERK-mediated phosphorylation of Elk-1 *in vitro*. Further, we show that either suppression of PADI4 or inhibition of PADI4 activity leads to reduced Elk-1 phosphorylation and histone H4 acetylation on the *c-Fos* promoter. These mechanistic studies support the hypothesis that PADI4 enzymatic activity potentiates two key features of *c-Fos* transcriptional activation; Elk-1 phosphorylation and histone acetylation.

## Results

### PADI4 Is Enriched near Gene Promoter Transcription Start Sites (TSSs)

The PADI4 antibody utilized for this study was selected because the immunogenic peptide sequence (N-terminal amino acids 1–15) is unique to PADI4 among the PADI family members (Figure S1A). In our study, an overexpressed Flag-tagged version of PADI4 was readily detected in MCF-7 cell extracts by western blot using both the anti-PADI4 antibody and an anti-Flag antibody, indicating accuracy of PADI4 antibody targeting. Furthermore, this antibody recognized PADI4 in the lysates of MCF-7 cells that had been transfected with PADI4 expression vector, but not in cells transfected with any other PADI family members, confirming antibody specificity (Figure S1B and S1C). Additionally as shown in Figure S2, ChIP analysis found that the PADI4 antibody also bound to the previously reported PADI4 targets, *OKL38* and *p21*.

**Figure 1:**
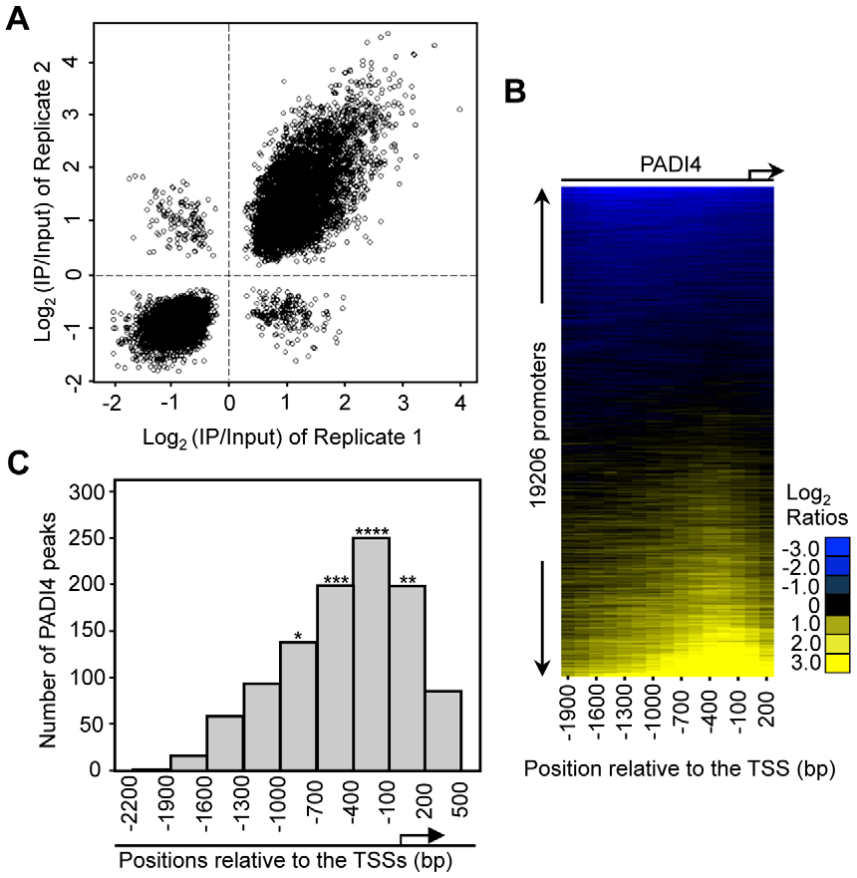
PADI4 is enriched in the gene promoter region near the TSSs. (A) Scatterplot showing the mean log_2_ ratio of each window on the array for two PADI4 ChIP-chip replicates. The spearman rank correlation coefficient and associated p-value are indicated. (B) Heat map of PADI4 ChIP-chip data for 19,206 promoters from −2,200 bp to +500 bp relative to the TSS. The values in the x-axis represent the center of a 600 bp window with 150 bp steps relative to each TSS. The promoters are ordered top to bottom based on the increasing intensity of the PADI4 signal at the promoter surrounding the TSSs. (C) Histogram showing the number of statistically significant peaks (Wilcoxon signed-rank test, p<0.016) across the entire tiled region for all the promoters on the ChIP-chip array. The asterisk denotes significant enrichment of PADI4 peaks near the TSSs based on the p-value from a Fisher exact test (*p = 0.0444, **p = 0.0085, ***p = 6.21×10^−9^, ****p = 1.42×10^−15^).

To identify PADI4 targets in an unbiased genome-wide manner, we performed ChIP in MCF-7 cells using the PADI4-specific antibody and then hybridized the enriched genomic DNA to promoter tiling arrays which spanned 2.7 kb of the gene promoter regions (2.2 kb upstream and 500 bp downstream relative to the TSS). The array represented 51 Mbp of genomic DNA, including nearly all known well-characterized RefSeq genes (19206 promoters total). The raw ChIP-chip signal to input ratios were processed as described in Materials and Methods. Significant PADI4 peaks were defined as the center of three consecutive windows with positive means (using at least six probes) and the center window having a mean greater than either adjacent window. The P-values for all the peaks were calculated using the nonparametric Wilcoxon signed-rank test. A total of 1124 peaks of PADI4 genomic binding were identified using a threshold P-value of less than 0.016 (Table S1). The use of this selection criterion was validated by conventional ChIP-qPCR analyses on the randomly selected peaks from the array (Figure S3) which showed a false positive rate (FPR) of less than 15%. As demonstrated by the strong correlation between the window averages from the ChIP-chip replicates (Figure 1A), the ChIP-chip results were found to be highly reproducible. To match the identified peaks with all well-characterized RefSeq genes, we examined the distribution of PADI4 peaks throughout the whole promoter region and aligned the peaks to the TSSs for all the genes within the array. Surprisingly, our heat map analysis showed an apparent enrichment of PADI4 in the region surrounding the TSS (Figure 1B). We next determined distribution of the distances of each significant PADI4 peak to the closest TSS and observed that the PADI4 peaks are statistically enriched around the TSSs (Figure 1C), suggesting that PADI4 may be involved in transcription factor mediated gene expression.

### PADI4 Levels Surrounding TSSs Are Positively Correlated with Gene Expression

**Figure 2:**
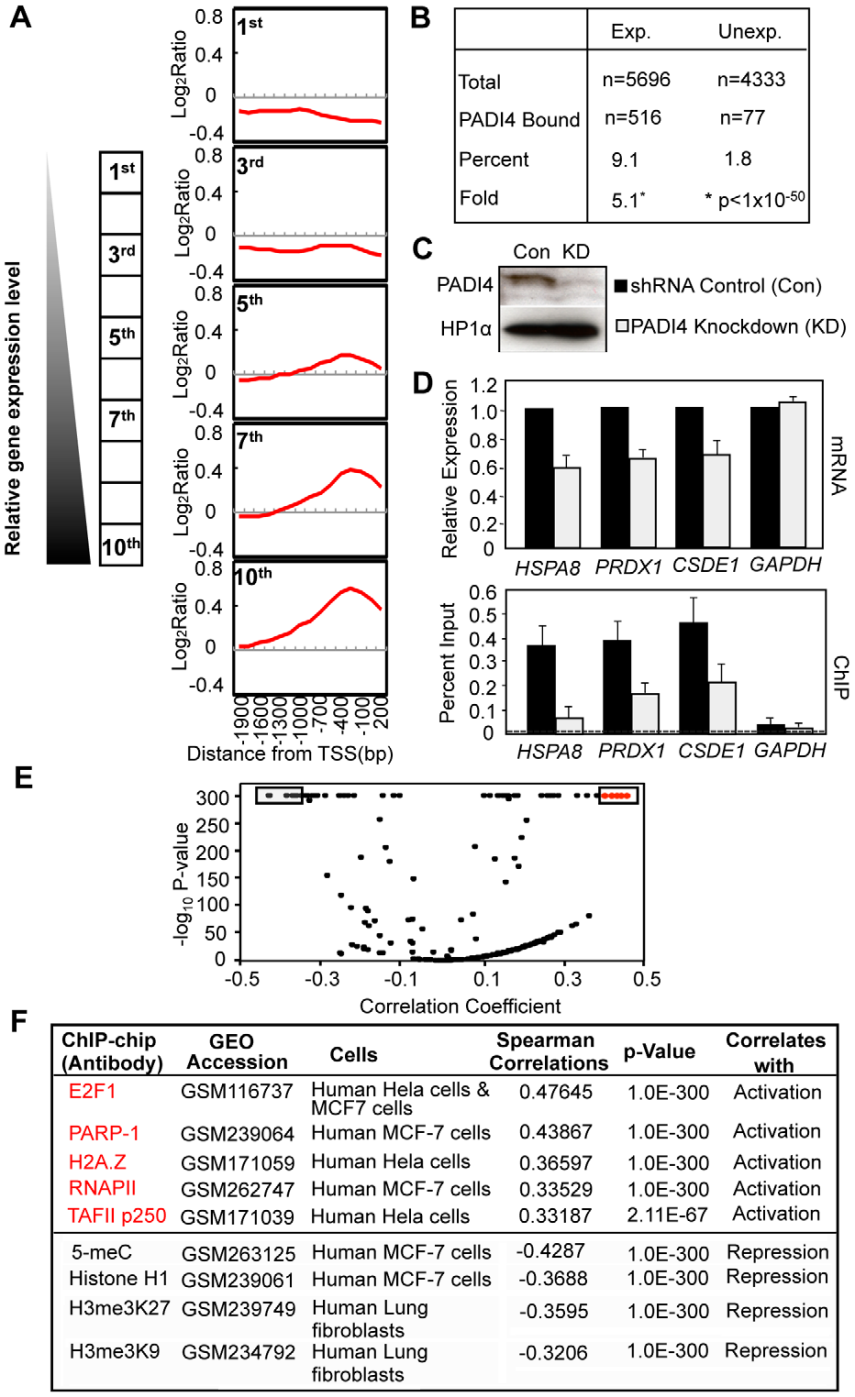
PADI4 recruitment at gene promoters correlates with the actively transcribed genes. (A) The average log_2_ enrichment ratio from PADI4 ChIP-chip is displayed for the genes in the MCF-7 cell cDNA microarray by decile of expression level from lowest (1^st^) to highest (10^th^). (B) Percentage and fold enrichment of PADI4-bound expressed and unexpressed genes. (C) Western Blot showing the shRNA-mediated depletion of PADI4 in MCF-7 cells versus shRNA control knockdown cells. (D) Gene-specific analysis of mRNA expression by RT-qPCR and PADI4 promoter binding by ChIP-qPCR in shRNA control and PADI4 knockdown MCF-7 cells. Expression data are normalized to β-actin transcripts and the graph is mean + SEM. The non-specific binding in the ChIP assay is indicated by the horizontal dotted line. (E) Volcano plot of computational comparison of PADI4 ChIP-chip dataset with multiple published ChIP-chip datasets. The score of each correlation was defined as the negative of the log_10_ of the p-value for the correlation. (F) PADI4 ChIP-chip is positively correlated with the ChIP-chips assayed by gene activating markers (in red) while negatively correlated with the ChIP-chips assayed by gene silencing markers (in black). The data shown here represent a subset of the data shown in Figure 2E.

To explore the relationship between PADI4 and gene expression in more detail, we compared our PADI4 ChIP-chip data sets with gene expression microarray analyses from MCF-7 cells that had been grown under similar conditions (Affymetrix expression arrays, U133A, GEO accession number GSE9253). We first plotted the average signal intensities for PADI4 around the TSSs for all of the genes in the expression microarray following sorting of these genes into ten distinct expression level subsets by decile (1^st^ being lowest 10% and 10^th^ being highest 10%). As shown in Figure 2A, most genes with either low or no expression (1^st^ decile) are not bound by PADI4. The average PADI4 ChIP-chip signal is then seen to increase around the TSS concordant with increasing levels of gene expression, with the 10^th^ decile showing the highest level of PADI4 binding. We then grouped all of the expressed genes (5696) in the microarray and identified 516 genes (9.1%) as having significant PADI4 peaks (p<0.016, Wilcoxon signed-rank test) while only 77 (1.8%) of the 4333 unexpressed genes showed significant PADI4 peaks, thus indicating a 5.1-fold enrichment of PADI4 at expressed genes (Fisher's Exact Test, p<1×10^−50^) compared to unexpressed genes (Figure 2B). This result supports a positive role of PADI4 in gene transcription. To gain further insight into the biological importance of the PADI4-bound genes, we determined the ontological gene categories by gene function analysis using DAVID (See Functional classification and annotation of the candidate genes, Genomic data analyses). As shown in Table 1 and Table S2, these genes encode proteins that are involved in a range of processes, with some of the most significant enrichment scores being for genes associated with membrane and organelle lumens, the nuclear lumen, protein catabolism, RNA processing, nucleotide binding, chromatin function, and ribosomal biogenesis. Next, we tested for a functional link between PADI4 and gene expression by evaluating the expression of a subset of PADI4-bound genes in both control MCF-7 cells and in stable short hairpin RNA (shRNA)-mediated PADI4 knockdown MCF-7 cells (Figure 2C and 2D). For each gene, we tested both gene expression by reverse transcription (RT)-qPCR and PADI4 promoter binding by ChIP-qPCR. Genes with reduced expression in the PADI4 knockdown also showed decreased PADI4 promoter binding while the expression of the *GAPDH* housekeeping gene (which is not bound by PADI4 at its promoter) remained unchanged in the PADI4 knockdown. This analysis further confirms the hypothesis that PADI4 binding is well correlated with transcriptional activation.

**Table 1:** Gene ontology analysis for the transactive genes associated with PADI4 binding in MCF-7 cells.

Gene ontology term	Aspect	P-value
membrane-enclosed lumen	CC	1.66E-12
intracellular organelle lumen	CC	3.43E-11
organelle lumen	CC	5.00E-11
nuclear lumen	CC	1.69E-09
ribonucleoprotein complex	CC	7.50E-09
non-membrane-bounded organelle	CC	1.35E-07
intracellular non-membrane-bounded organelle	CC	1.35E-07
macromolecule catabolic process	BP	3.11E-11
cellular macromolecule catabolic process	BP	4.88E-11
modification-dependent macromolecule catabolic process	BP	1.19E-09
modification-dependent protein catabolic process	BP	1.19E-09
proteolysis involved in cellular protein catabolic process	BP	1.57E-09
protein catabolic process	BP	1.62E-09
cellular protein catabolic process	BP	1.83E-09
nucleotide binding	MF	1.37E-07
ATPase activity	MF	6.05E-06
purine nucleotide binding	MF	2.22E-05
ribonucleotide binding	MF	2.62E-05
purine ribonucleotide binding	MF	2.62E-05
RNA binding	MF	3.89E-05
nucleoside binding	MF	1.14E-04

The analysis was performed for all three gene ontology aspects: cellular component (CC), biological process (BP) and molecular function (MF). The seven most significant gene ontology terms for each aspect are listed. For a more complete list of significantly enriched ontologies, refer to Table S2.

To further test the hypothesis that PADI4 promoter binding correlates with transcriptional activation, we next compared our PADI4 ChIP-chip dataset with multiple published ChIP-chip datasets for factors that are well correlated with gene regulation (http://www.ncbi.nlm.nih.gov/geo) [21]. The spearman correlation was determined between windows of the PADI4 ChIP-chip and identical windows within each of ChIP-chip data sets incorporated in the database. Volcano plots showed both statistically significant positive (Figure 2E, on the right) and negative (Figure 2E, on the left) correlations between the published ChIP-chip datasets and our PADI4 dataset. For example, as shown in Figure 2F, the PADI4 ChIP-chip output is significantly correlated with output from factors associated with gene activation such as the transcription factors E2F1 and RNAPII [22]–[25], the chromatin modulating protein PARP-1 [26] and the histone varaiant H2A.Z [27]. On the other hand, the PADI4 ChIP-chip dataset is negatively correlated with datasets from factors associated with gene repression such as the H3me3K9 and H3me3K27 histone modifications and methylated DNA (5-meC) [13], [28], [29]. Taken together, this computational analysis again suggests that PADI4 binding plays a role in the activation of a subset of actively transcribed genes.

### Combinatorial Promoter Targeting by PADI4 and Active Elk-1

**Figure 3:**
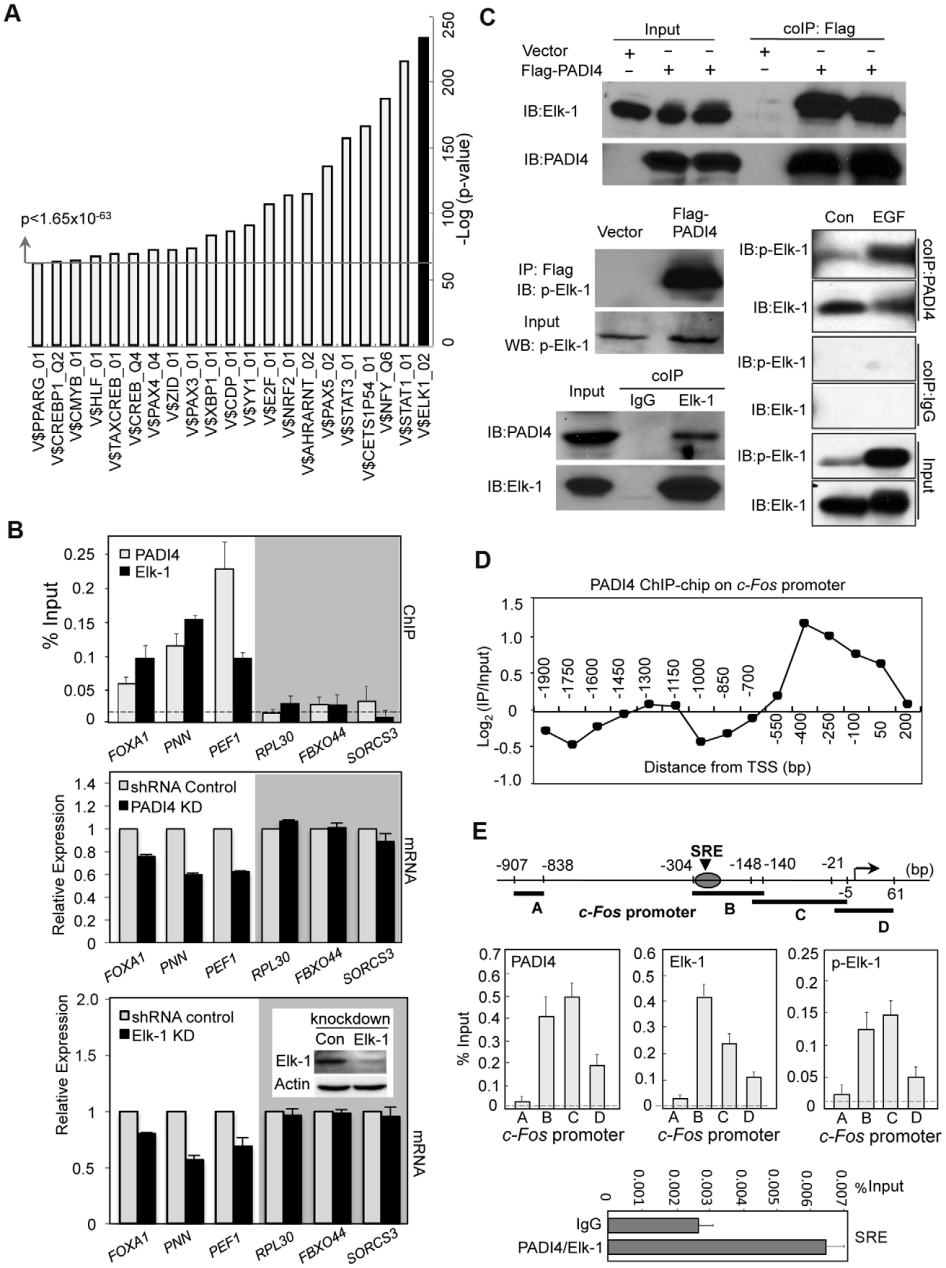
Co-occupancy by PADI4 and Elk-1 on a subset of Elk-1 target gene promoters. (A) Identification of enriched TFBS within the promoter regions of the significant PADI4 bound genes (p<0.016). The bar chart displays the negative log of the enrichment P values using Fisher exact test for the top 21 motifs. The most significant V$ELK1_02 is shown on the right in black. (B) Gene-specific analysis of promoter binding by PADI4 and Elk-1 using ChIP-qPCR in MCF-7 cells (upper panel) and mRNA expression by RT-qPCR in shRNA control and PADI4 knockdown (middle panel) or Elk-1 knockdown MCF-7 cells (lower panel). The insert in the lower panel shows the shRNA-mediated depletion of Elk-1 in MCF-7 cells versus shRNA control cells by western blot. For each gene, the independent PADI4 and Elk-1 ChIP experiments were performed with the same promoter primers. The non-specific binding in the ChIP assay is indicated by the horizontal dotted line. Expression data are normalized to *GAPDH* transcripts and the graph is mean + SEM. The control genes without potential binding sites for both PADI4 and Elk-1 on the promoter are shown in the gray shadow. (C) Co-IP assays in 293 cells reveal that PADI4 interacts with both Elk-1 and phospho-Elk-1. Lysates of HEK293 cells transfected with a plasmid carrying Flag-PADI4 were immunoprecipitated with anti-Flag M2 affinity gel followed by western blot analysis using antibodies against Elk-1, phospho-Elk-1 and PADI4 (upper and middle left). Endogenous Elk-1 proteins were immunoprecipitated with anti-Elk-1 antibody, and co-precipitated PADI4 proteins were subsequently detected with anti-PADI4 antibody (bottom left). Immunoblot on the bottom right shows increased levels of p-Elk-1 binding to PADI4 following stimulation with EGF. HEK293 cells were transiently transfected with PADI4, serum starved, and stimulated with EGF. Immunoprecipitations of cell lysates was then performed using the anti-PADI4 antibody and immunoblots were then probed with both anti-p-Elk-1 and anti-Elk-1. Normal IgG antibody was used as a control. (D) PADI4 ChIP-chip signal localizes to the proximal *c-Fos* promoter. (E) ChIP-qPCR showing that PADI4 and activated Elk-1 specifically bind at the *c-Fos* SRE promoter region in MCF-7 cells. The schematic at the top of the figure indicates the locations of the primer sets used for ChIP assays relative to the TSS. The SRE region is marked. The non-specific binding in the ChIP assay is indicated by the horizontal dotted line. Data are represented as mean + SEM (n = 3). ChIP-re-ChIP experiments (bottom) detected simultaneous binding of PADI4 and Elk-1 to the *c-Fos* SRE region in MCF-7 cells. The first step ChIP was performed using anti-PAD4 antibody and the second step ChIP was performed using anti-Elk-1 antibody. IgG was used as an irrelevant control.

To identify specific transcription factors that might utilize PADI4 to mediate transcriptional output, we computationally explored the promoter regions of genes with significant PADI4 binding (p<0.016) to identify putative DNA binding motifs within the bound regions (See Enrichment of Transcription Factor Binding Sites [TFBS] Analysis, Genomic data analyses). The analysis identified a number of highly enriched DNA binding elements for transcription factors such as STAT1/3, NFY, and E2F, suggesting the possible involvement of these *cis*-elements in the underlying regulatory control of PADI4 at target genes (Figure 3A and Figure S4). The transcription factor binding site showing the highest level of significance (Fisher exact test, p = 5.56E-235) was V$ELK1_02, which contains the consensus motif CCGGAA. This motif was originally identified as a binding site for the oncogene, Elk-1, and has since been found to be bound by other ETS family members such as Ets1 and GABPα [30]–[32]. Interestingly, of the 516 gene promoters showing significant PADI4 binding, 70 genes (13.56%) were found to also contain the V$ELK1_02 site adjacent to, or overlapping PADI4 peaks. Further, a ChIP-chip data set is publically available for Elk-1 [33] and a comparison of our PADI4 dataset with this data showed that 15.7% of PADI4 peaks are found within 1.5 kb of Elk-1 peaks (Table 2), suggesting that Elk-1 is significantly enriched near PADI4 peaks. This observation suggested that Elk-1 may utilize PADI4 as a cofactor to help mediate transcriptional activation.

**Table 2:** Comparison of PADI4 ChIP-chip with Elk-1 ChIP-chip dataset [33].

Test factor	Co-occupied peaks	Only test	Only PADI4	Fold enrichment	p-value	% of test	% of PADI4	Cell line
Elk-1	119	367	638	55.4	3.36E-147	24.5	15.7	Serum-starved Hela Cells

To begin testing this hypothesis experimentally, we performed independent PADI4 and Elk-1 ChIP experiments using identical promoter primers to test whether Elk-1 and PADI4 bound to the promoters of a subset of genes identified as having PADI4 peaks and the V$ELK1_02 element. Results show that, as predicted, both PADI4 and Elk-1 bound to the same promoter regions of this subset and not to the promoters of a subset of control genes identified as both PADI4 and V$ELK1_02 negative (Figure 3B). To test if PADI4 and Elk-1 might coregulate the expression of this bound subset, we generated a stable Elk-1 knockdown MCF-7 cell line (Figure 3B, bottom panel, inset) and then evaluated the expression of the bound and unbound genes in the Elk-1 and PADI4 knockdown lines. We found that PADI4 or Elk-1 depletion in MCF-7 cells reduced the expression of the bound genes while expression of unbound genes (shaded in gray) remained unchanged (Figure 3B). These results support the hypothesis that Elk-1 may utilize PADI4 as a co-factor to regulate gene expression.

To further test this hypothesis, we next carried out co-immunoprecipitation analysis to establish whether Elk-1 and PADI4 interact. We first confirmed the specificity of the anti-Elk-1 antibody in HEK293 cells by depleting Elk-1 and showing that levels of the anti-Elk-1-reactive band were greatly reduced (Figure S5). Next, we transiently transfected Flag-tagged PADI4 into 293 cells and immunoprecipitated with anti-Flag antibody followed by western blot analysis using antibodies against both Elk-1 and phospho-Elk-1 (p-Elk-1). As shown in Figure 3C, a substantial amount of Elk-1 and p-Elk-1 was co-precipitated from cells expressing Flag-PADI4, but not from cells lacking Flag-PADI4. Additionally, we also found that levels of p-Elk-1 binding to PADI4 increased following stimulation with EGF (Figure 3C). Reciprocally, we also found that the endogenous Elk-1 proteins were immunoprecipitated with anti-Elk-1 antibody, and the co-precipitated PADI4 proteins were subsequently detected using the anti-PADI4 antibody, while PADI4 was not coimmunoprecipitated with the normal rabbit IgG control (Figure 3C). These results indicated that both Elk-1 and the active form of Elk-1 can physically associate with PADI4. The interaction of PADI4 and phosphorylated Elk-1 supports the hypothesis that PADI4 may play a direct role in facilitating Elk-1 target gene activation.

Perhaps the best-characterized Elk-1 target is the immediate early oncogene *c-Fos*. The SRE *cis* element that mediates *c-Fos* transcriptional activation contains the Elk-1 binding element and is recognized by the Elk-1-containing ternary complex factor [34]. This observation prompted us to examine whether the location of PADI4 on the *c-Fos* promoter (as determined by ChIP-chip) is near the SRE region. Interestingly, PADI4 binding was found to primarily occur in the proximal promoter region of *c-Fos*, covering the SRE motif (Figure 3D and 3E). This result implied that PADI4 and Elk-1 may also target the same promoter region of *c-Fos*. Therefore, we further characterized this interaction on the tiled regions of the *c-Fos* promoter by performing PADI4, Elk-1 and p-Elk-1 ChIPs in MCF-7 cells (Figure 3E). In line with our hypothesis, we found endogenous PADI4 to be enriched specifically around the SRE motif on the *c-Fos* promoter but not on promoter regions more removed from the TSS (∼500 bp upstream of the SRE or downstream of the TSS). We also observed a near perfect overlap between PADI4, p-Elk-1, and Elk-1 at the *c-Fos* promoter by ChIP q-PCR. Given these observations, and that PADI4 interacts with Elk-1 in 293 cells, we also carried out ChIP-re-ChIP analysis using anti-PADI4 antibody for the first round of IP and then anti-Elk-1 for the second round of IP. Results showed that endogenous PADI4 appears to physically associate with Elk-1 at the *c-Fos* SRE region (Figure 3E, bottom). Given these observations, we next decided to more closely investigate the role of PADI4 in *c-Fos* regulation to gain mechanistic insight into PADI4's role in transcriptional activation.

### PADI4 Mediates Expression of the Elk-1 Target Gene *c-Fos*

**Figure 4:**
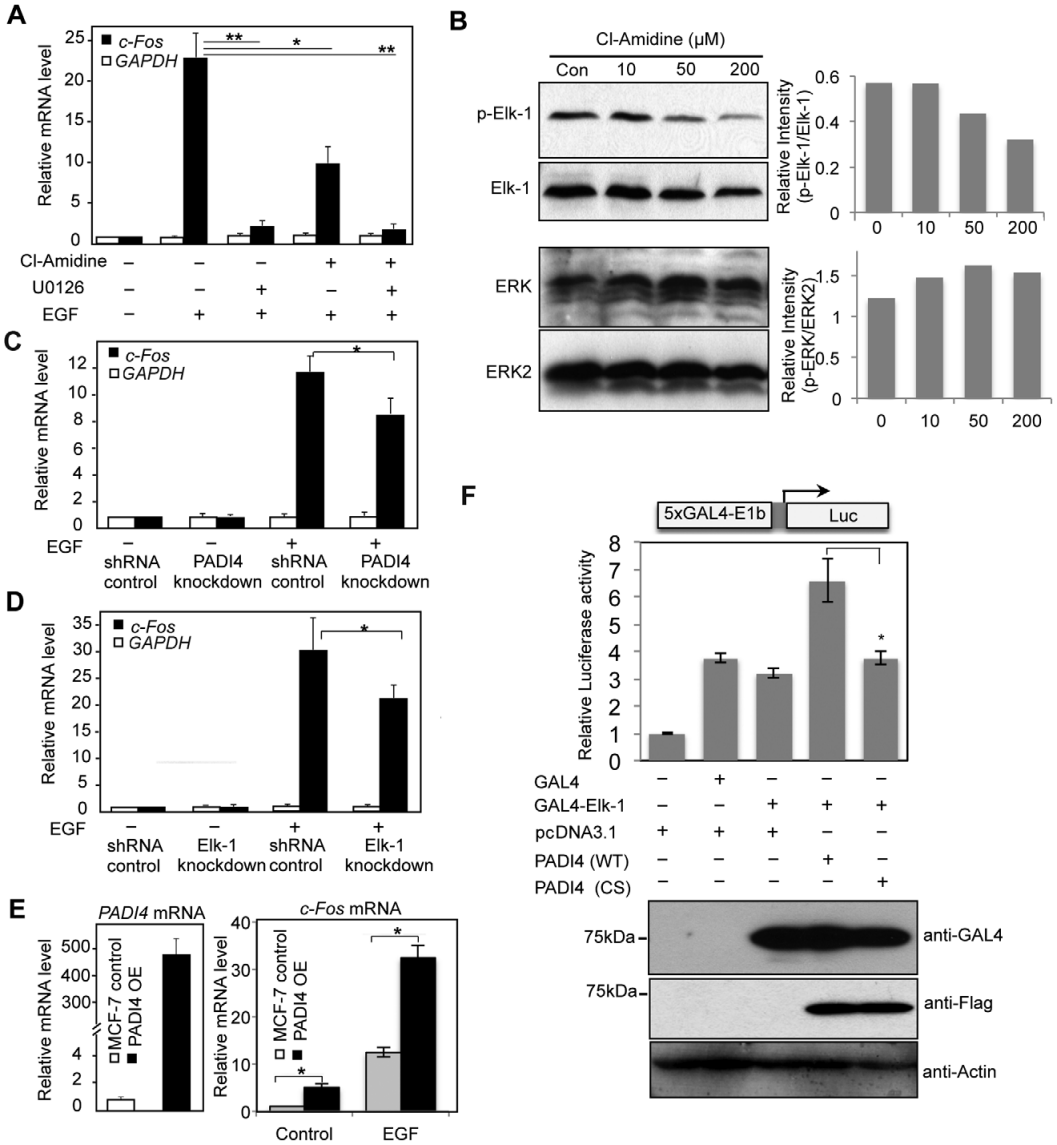
PADI4 mediates expression of the Elk-1 target gene *c-Fos*. (A, C and D) Real-time RT-PCR analysis of the *c-Fos* and *GAPDH* expression in serum-starved or EGF-stimulated MCF-7 cells (A) with or without Cl-Amidine or U0126 treatment; (C) with or without PADI4 knockdown; (D) with or without Elk-1 knockdown. Expression data are normalized to β-actin transcripts and the graph represents the mean + SEM (n = 3). *P<0.05, **P<0.01. (B) Dose-dependent effect of Cl-Amidine treatment on Elk-1 activation and ERK2 phosphorylation in MCF-7 cells. Different doses of Cl-Amidine were added to the normal MCF-7 cell culture media for 48 hours and western blots of cell lysates were then performed using anti-p-Elk-1, anti-Elk-1, anti-p-ERK and ERK2 antibodies. The graph on the right is a semi-quantitative analysis of the western blots (relative intensity) with an arbitrary number of the intensity ratio of p-Elk-1 compared to total Elk-1, or p-ERK compared to total ERK2 using Image J software. (E) Stable overexpression (OE) of PADI4 in MCF-7 cells increases transcriptional output of *c-Fos*. Left: qRT-PCR analysis showing that *PADI4* expression is increased in PADI4 OE MCF-7 cells. Right: qRT-PCR analysis showing that *c-Fos* transcription output was significantly elevated in PADI4 OE MCF-7 cells under either control or EGF treatment. (F) PADI4 enzymatic activity facilitates Elk-1 mediated gene transcription. Expression vector for the DNA-binding domain of GAL4 (CMV-GAL4) or GAL4-Elk-1 was transfected into HEK293 cells along with the 5xGAL4-driven luciferase reporter plasmid containing the minimal E1b promoter and plasmids for pcDNA3.1, wild type (WT) or inactive mutant (C645S) PADI4 with N-terminal Flag tags. Luciferase activity was determined per microgram total cell protein 24 hours after transfection and results are represented as fold relative to empty vector control. Data shown are representative of two independent experiments performed in triplicate and error bars indicate SEM. *P<0.05 (two-tailed paired Student's t-test). In the lower panel, the expression of Flag-PADI4 and GAL4-Elk-1 fusion proteins was monitored by western blot using anti-Flag and anti-GAL4 antibodies.

EGF can activate *c-Fos* expression in MCF-7 cells [35] and this response is accompanied by activation of Elk-1 via MAPK/ERK phosphorylation, by which the SRE *cis* element that mediates *c-Fos* transcriptional activation is recognized [8], [10], [11]. Given our finding that PADI4 and active Elk-1 strongly co-occupy the *c-Fos* promoter, we hypothesized that *c-Fos* expression may be regulated, in part, by the interplay between PADI4 and Elk-1. To address this question, we first tested the effects of inhibition of either Elk-1 or PADI4 activity on *c-Fos* expression following EGF stimulation. We inhibited PADI4 enzymatic activity using a newly developed arginine-based PADI inhibitor, Cl-Amidine [36], which has been shown to inhibit PADI4 activity at gene promoters in several cancer cell lines [19], [20]. As shown in Figure 4A, stimulation of MCF-7 cells with EGF (50 ng/ml) for 30 min elicited a dramatic increase of *c-Fos* mRNA expression. As expected, pretreatment of MCF-7 cells with the ERK inhibitor U0126 abrogated *c-Fos* activation following EGF stimulation. Interestingly, Cl-Amidine also strongly inhibited *c-Fos* activation by EGF (Figure 4A) and this inhibition was found to be dose-dependent (Figure S6). We also observed by western blotting that Cl-Amidine globally suppresses Elk-1 phosphorylation in a dose-dependent manner, while having little effect on ERK phosphorylation (Figure 4B). These observations suggests that the effect of Cl-Amidine on *c-Fos* transcription occurs downstream of ERK kinase, likely at the level of Elk-1 phosphorylation. Note: Given the strong suppressive effect of U0126 in *c-Fos* expression, we were not able to quantitatively test for possible synergy between this drug and Cl-Amidine. To control for non-specific effects of the drugs on transcription, levels of *GAPDH* housekeeping gene were also monitored and neither inhibitor altered the expression of this gene. As another test of the collaborative role of PADI4 and Elk-1 in *c-Fos* activation, we investigated *c-Fos* expression following EGF stimulation in the Elk-1 and PADI4 knockdown lines. As predicted, depletion of either PADI4 or Elk-1 reduced *c-Fos* expression upon EGF stimulation (Figure 4C and 4D). We then generated a stable PADI4-overexpressing line and found *c-Fos* expression to be elevated in this line in both un-stimulated and EGF stimulated conditions (Figure 4E). We also tested whether the observed inhibitory effect of Cl-Amidine on *c-Fos* transcription is preserved in other breast cancer cell lines by treating BT474 (ER+, HER2+) and MCF10A-DCIS (ER−, HER2+) cells with the inhibitor. Results showed that Cl-Amidine treatment inhibits EGF-induced *c-Fos* expression in both of these lines, with the strongest inhibitory effect being observed in the BT474 line (Figure S7).

**Figure 5:**
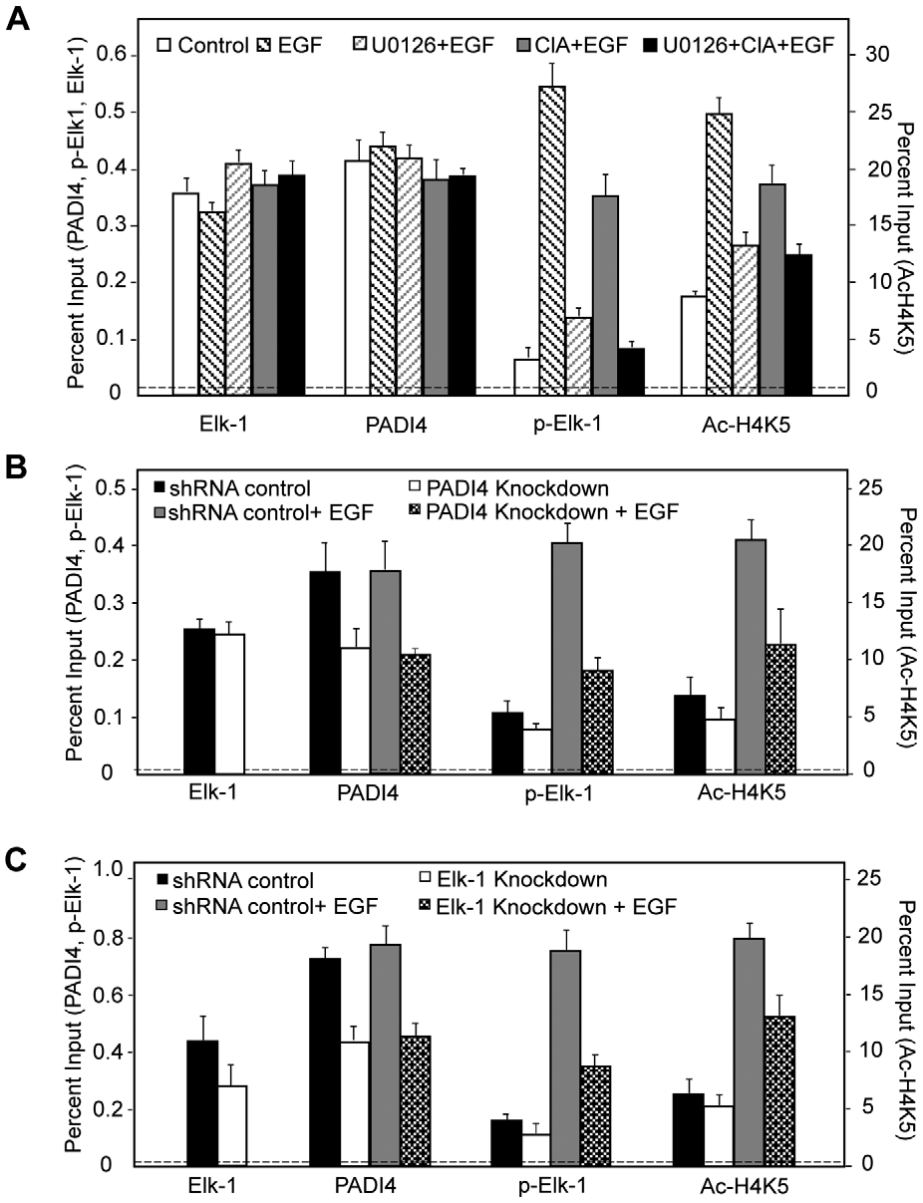
PADI4 enzymatic activity facilitates *c-Fos* transcription via regulation of Elk-1 phosphorylation and/or HAT-mediated histone acetylation. (A–C) ChIP-qPCR analysis of PADI4, Elk-1, p-Elk-1, and Ac-H4K5 binding at *c-Fos* promoter in serum-starved or EGF-stimulated MCF-7 cells; (A) in the absence or presence of Cl-Amidine or/and U0126 treatment; (B) with or without PADI4 knockdown; or (C) with or without Elk-1 knockdown. The IgG control in the ChIP assay is indicated by the horizontal dotted line.

We next investigated whether PADI4 and Elk-1 might coregulate gene activation on a more generalized template *in vitro*. For this experiment, we fused full length Elk-1 to the GAL4 DNA-binding domain, and transfected this construct alone or with either WT PADI4 or the PADI4 catalytic mutant (CS) into 293 cells. We then tested the effects of these constructs on GAL4-driven luciferase reporter activity. Results showed that while GAL4-Elk-1 did not activate transcription over GAL4 alone, cotransfection of WT PADI4 with Elk-1 resulted in enhanced luciferase activity (Figure 4F). PADI4 enzymatic activity appears to be required for reporter activity because cotransfection of GAL4-Elk-1 with inactive PADI4 did not increase reporter activity above that of Elk-1 alone. We also carried out luciferase reporter experiments using a *c-Fos*-luciferase reporter system and obtained similar results (Figure S8). Collectively, the above data support the hypothesis that a functional relationship between Elk-1 and PADI4 on gene promoters potentiates Elk-1 mediated gene transcription. To further test this hypothesis and to investigate whether Elk-1 recruits PADI4 to its target promoters (or possibly vice versa), we then carried out ChIP-qPCR analysis with PADI4 and Elk-1 antibodies in serum-starved MCF-7 cells, in cells treated with EGF, and in EGF-stimulated cells that had been pretreated with inhibitors. Results showed that binding of PADI4 and Elk-1 at the *c-Fos* SRE promoter region was independent of EGF, U0126 or Cl-Amidine treatment (Figure 5A). Additionally, we found that PADI4 occupancy was decreased at the *c-Fos* SRE region upon PADI4 knockdown, while depletion of PADI4 did not change Elk-1 occupancy at *c-Fos* (Figure 5B). On the other hand, depletion of Elk-1 not only caused a decrease in Elk-1 at the *c-Fos* promoter but also reduced PADI4 enrichment at this site (Figure 5C). The binding of Elk-1 at *c-Fos* promoter before and after EGF stimulation is consistent with previous reports showing that Elk-1 constitutively binds to *c-Fos* promoter [9], [37]. Given that PADI4 has no distinguishable DNA binding motif and that PADI4 has been found to be targeted to specific gene promoters by transcription factors such as p53 [19], our results suggest that PADI4 recruitment to the *c-Fos* promoter is Elk-1-dependent.

In order to further test how PADI4 regulates *c-Fos* activity, we next tested the effects of PADI4 inhibition or depletion on Elk-1 phosphorylation and on histone H4K5 acetylation (Ac-H4K5) at the *c-Fos* SRE following EGF stimulation in MCF-7 cells (Figure 5). Results showed that EGF treatment resulted in a strong enrichment of p-Elk-1 and Ac-H4K5 at the *c-Fos* promoter (Figure 5A) and that this enrichment was abrogated by pretreatment with the PADI4 inhibitor (Figure 5A) or by PADI4 depletion (Figure 5B). Additionally, as expected, both ERK inhibition (U0126, Figure 5A) and Elk-1 depletion (Figure 5C) also reduced p-Elk-1 and Ac-H4K5 enrichment at *c-Fos* promoter following EGF stimulation.

### Citrullination of Elk-1 by PADI4 Potentiates Elk-1 Phosphorylation

**Figure 6:**
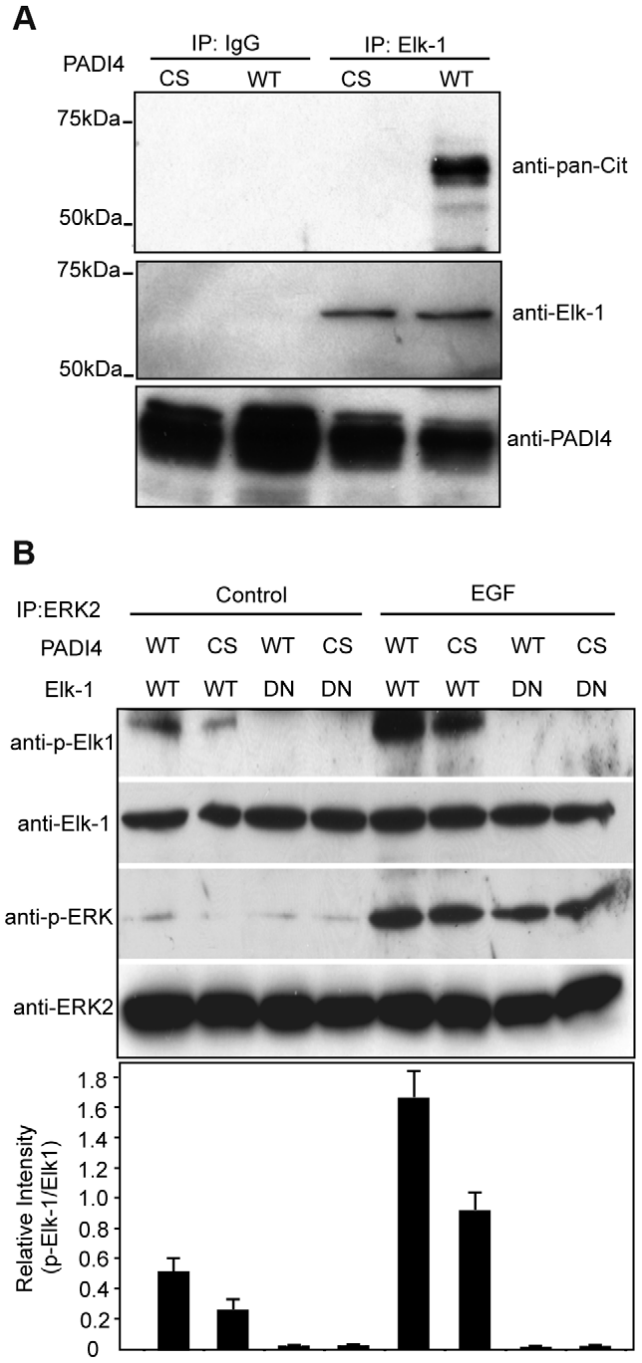
Citrulliantion of Elk-1 by PADI4 potentiates Elk-1 phosphorylation. (A) Citrullination of Elk-1 by enzymatically active PADI4 *In vitro*. Elk-1 protein from MCF-7 cells was immunoprecipitated by anti-Elk-1 antibody and then treated with either WT or C645S mutant PADI4 purified from the Ni-NTA column. The reactions were assessed by western blot analysis with an antibody that recognizes citrullinated proteins (anti-pan-citrulline antibody). IgG was used as internal control. (B) *In vitro* citrullination of Elk-1 by active PADI4 facilitates the activation of Elk-1 by ERK2 kinase. JEG3 cells were serum starved and then treated with 50 ng/ml EGF for 30 min. The cells were lysed and ERK2-containing proteins were immunoprecipitated with anti-ERK2 antibody. His-tagged PADI4 (WT or C645S catalytic inactive mutant) purified from the Ni-NTA column was used to treat the *in vitro* translated Elk-1 (WT or DN mutant). The PADI4-treated Elk-1 was then incubated with immobilized ERK2 for the kinase assay. The products were detected by Western blotting with antibodies to p-Elk-1, Elk-1, p-ERK, and ERK2. The graph below indicates densitometric analysis of western blots (relative intensity) with an arbitrary number of the intensity ratio of p-Elk-1 to total Elk-1 using Image J Software.

The findings that PADI4 interacts with Elk-1 on *c-Fos* and that PADI4 inhibition suppressed phosphorylation of Elk-1 (but not ERK) suggested that PADI4 may regulate *c-Fos* activation by directly targeting Elk-1 for citrullination. To test the hypothesis, we first investigated whether PADI4 can citrullinate Elk-1 *in vitro*. Endogenous Elk-1 was immunoprecipitated from MCF-7 cells, treated with recombinant PADI4 (WT or CS), and the resolved proteins were then probed with an antibody that is reactive with citrullinated proteins (anti-pan-cit, [38]). Results (Figure 6A) show that the anti-pan-cit antibody was reactive with an appropriately sized band from the anti-Elk-1 immunoprecipitate (lane 4) and was not reactive with proteins from the anti-Elk-1 immunoprecipitate (lane 3) that had been treated with the PADI4 C645S mutant or from proteins immunoprecipitated with IgG (lanes 1 and 2). Anti-Elk-1 and anti-PADI4 western blots confirmed equal protein loading. These results support the hypothesis that Elk-1 is a target for PADI4 mediated citrullination.

Transcriptional activation of *c-Fos* is initiated following phosphorylation of Elk-1 by ERK kinase. The above findings raised the possibility that citrullination of Elk-1 by PADI4 may facilitate ERK-mediated phosphorylation of Elk-1. To test this hypothesis, we then performed ERK kinase assays on *in vitro* translated WT or inactive mutant (DN) Elk-1 following pretreatment with either WT or catalytically inactive (CS) PADI4. As shown in Figure 6B, in the presence of activated ERK2, higher levels of Elk-1 Ser383 phosphorylation were observed following pretreatment of Elk-1 with WT PADI4 (lane 5) when compared to pretreatment with inactive PADI4 (lane 6). A similar decrease in Elk-1 Ser383 phosphorylation levels was observed with the ERK2 isolated from the non-EGF stimulated JEG3 cells following treatment of Elk-1 with mutant PADI4, though the overall signal intensities were much lower (lanes, 1 and 2). As expected, phosphorylation of DN Elk-1 was below the limit of detection (lanes 3, 4, 7 and 8), thus confirming anti-p-Elk-1 antibody specificity. Total Elk-1 levels were also evaluated with anti-Elk-1 antibody to normalize for protein loading. Additionally, the blots were also probed with anti-phosphorylated ERK antibody to confirm that EGF treatment of JEG3 cells activated ERK2 and with anti-ERK2 antibody to further confirm equal protein loading. Collectively, these results support the hypothesis that PADI4 mediated citrullination facilitates phosphorylation of Elk-1 by ERK.

## Discussion

Given previous findings indicating that PADI4 plays a repressive role in gene transcription, we were initially surprised by our global analysis showing that PADI4 binding is primarily correlated with active gene transcription. However, the prediction that PADI4 also appears to play an important role in the activation of a broad range of target genes is supported by the following new lines of evidence. Firstly, we found that the PADI4 binding pattern near the TSSs is highly correlated with actively transcribed genes in MCF-7 cells and that most genes not expressed in the array are not bound by PADI4. Secondly, our computational ChIP-chip dataset analysis found that PADI4 is highly positively correlated with datasets from factors associated with gene activation and negatively correlated with datasets from factors associated with gene repression. Lastly, our computational analysis of transcription factor binding sites found that PADI4 is significantly enriched on promoters which contain DNA binding elements for a broad range of activating transcription factors. Interestingly, several of the elements identified in our computational analysis are bound by transcription factors such as STAT1, STAT3, STAT5, ETS1, PAX and E2F, which play important roles in mammary function and in breast cancer [39]–[44]. Thus, the finding that PADI4 binding is enriched near these elements in MCF-7 cells supports the hypothesis that PADI4 plays a role in regulating target gene expression in a range of mammary gland signaling pathways.

Given that PADI4 binding was frequently coincident with the Elk-1 DNA binding element, and that PADI4 interacts with Elk-1, we next focused on investigating the potential relationship between PADI4 and Elk-1 on the *c-Fos* promoter, in order to better understand how PADI4 may activate gene transcription. Our findings that endogenous *c-Fos* levels were suppressed in PADI4 depleted and inhibitor-treated lines and elevated in PADI4 overexpressing cells, supported our genome-wide finding that PADI4 plays a role in transcriptional activation. Next, the observations that PADI4 interacts with Elk-1 at *c-Fos* and that PADI4 facilitates Elk-1-mediated activation of both a ubiquitous, and *c-Fos*-specific, reporter systems suggested that a functional relationship between Elk-1 and PADI4 confers activation of *c-Fos*. As to potential mechanisms behind this functional relationship, we found that PADI4 can citrullinate Elk-1 *in vitro* and that PADI4 enzymatic activity facilitates Elk-1 phosphorylation on *c-Fos*. Our ERK kinase assay then demonstrated that citrullination of Elk-1 by PADI4 enhances ERK-mediated Elk-1 phosphorylation. Finally, the observation that histone H4K5 acetylation was suppressed at *c-Fos* following PADI4 inhibition or depletion suggested that the observed PADI4 enzymatic activity on Elk-1 phosphorylation at *c-Fos* then facilitated histone acetylation and subsequent *c-Fos* activation.

**Figure 7:**
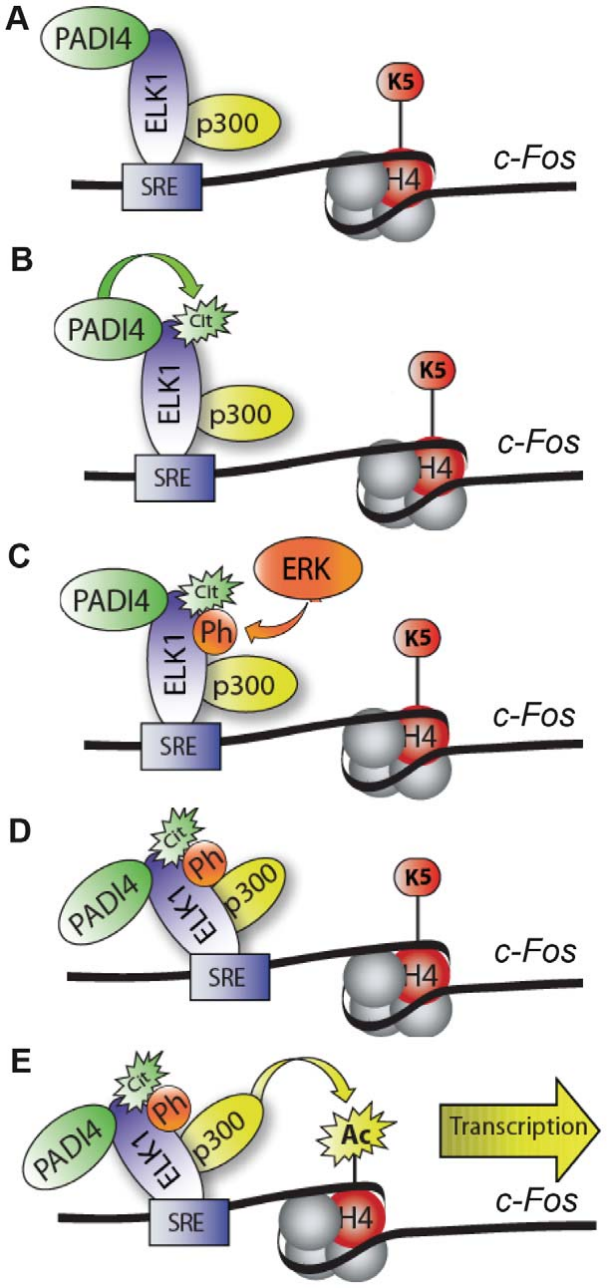
Working model depicting role of PADI4 in Elk-1–mediated activation of *c-Fos*. (A) Prior to stimulation, Elk-1, PADI4, and p300 constitutively occupy the *c-Fos* promoter. (B) Following EGF treatment, Elk-1 is citrullinated by PADI4. (C) Upon citrullination, Elk-1 is then phosphorylated by ERK2. (D) This phosphorylation event leads to an enhanced interaction between Elk-1 and p300 (9). (E) The enhanced interaction then potentiates p300-mediated histone acetylation and subsequent transcriptional activation.

The potential role for PADI4 in *c-Fos* activation is partially clarified when the above findings are put in the context of what is currently known about the role of Elk-1 in this process. Recent reports have demonstrated that unphosphorylated Elk-1 and p300 form complexes and bind to gene promoters in the absence of stimuli. Following stimulation, Elk-1 is then phosphorylated by ERKs and this phosphorylation event strengthens the interaction between Elk-1 and p300, leading to enhanced histone acetyltransferase activity at target promoters and gene activation [9], [14]. In addition to observed Elk-1/p300 interactions, p300 has also been found to be targeted by PADI4 for citrullination [45]. Given these observations, and our findings that PADI4 interacts with Elk-1 and that Elk-1 depletion reduces PADI4 levels at *c-Fos*, it seems likely that Elk-1, p300, and PADI4 are constitutively bound to the *c-Fos* promoter (Figure 7). Our current working model then predicts that treatment of MCF-7 cells with EGF then activates PADI4, which, in turn, citrullinates Elk-1. Upon citrullination, Elk-1 is then phosphorylated by ERK2 leading to stronger associations between Elk-1 and p300, increased histone acetylation, and subsequent transcriptional activation [4], [8], [10], [46]. Given that citrullination neutralizes positively charged arginine residues, it seems possible that PADI4-mediated citrullination facilitates ERK-mediated phosphorylation of Elk-1 by allowing this kinase closer access to its target serine residues. As an alternate hypothesis to our working model, Lee et al. [45] has found that PADI4 can also directly citrullinate p300 to enhance p300 activity. Thus, it is possible that a direct functional relationship exist between PADI4 and p300 on *c-Fos*. However, we found that treatment of p300 with PADI4 did not enhance p300's histone acetyltransferase activity *in vitro* (data not shown). Additionally, we do not believe that PADI4 is mediating *c-Fos* activity via histone citrullination as we were not able to detect significant increases of citrullinated histones (H3 or H4) on the *c-Fos* promoter after EGF stimulation (data not shown). Given these observations, and our findings presented here, the most likely mechanism by which PADI4 is regulating *c-Fos* is by targeting Elk-1 for citrullination.

In conclusion, our genomic analysis has identified a new role for PADI4 in mediating gene transactivation. We present evidence that a broad range of transcription factors may utilize PADI4 as a cofactor to mediate their transactivation functions, thus suggesting that the gene regulatory role for PADI4 is much broader than previously thought. We also provide new mechanistic insights into how PADI4 mediates gene transactivation by showing that PADI4 interacts with and citrullinates Elk-1 and that PADI4 enzymatic activity potentiates two key features of *c-Fos* activation, Elk-1 phosphorylation and histone H4 acetylation. Studies are currently underway to further dissect the mechanism by which PADI4 regulates gene expression and possibly mammary tumorigenesis.

## Materials and Methods

### Cell Culture and Constructs

MCF-7 cells were maintained in DMEM supplemented with 5% calf serum and both HEK293 and JEG3 cells were maintained in DMEM supplemented with 10% fetal bovine serum. Elk-1-depleted MCF-7 and 293 cell lines were generated by transduction with Mission Lentiviral Transduction Particles containing a short hairpin RNA (shRNA) construct targeting the human Elk-1 coding sequence (Sigma SHCLNV-NM_005229). In the control group, cells were transduced with a non-targeting shRNA lentiviral construct (Sigma SHC002V). Cells were selected by medium containing 1 µg/ml puromycin (Sigma). PADI4-depleted MCF-7 cells were generated by transfection with Mission shRNA Plasmid DNA targeting the human PADI4 coding sequence (Sigma SHCLND-NM_012387). Control cells harboring a non-targeting shRNA lentiviral construct (Sigma SHC002V) were generated in parallel. PADI4 overexpression MCF-7 cells were generated by transfection with PADI4-pcDNA3.1(+) and selected by medium containing 500 µg/ml G418 (Gibco). Where indicated, Cl-Amidine was diluted in cell culture medium at the final concentration of 200 µM and added to cells for 40 hr prior to harvest. Where indicated, cells were serum starved for 12 hr (expression analysis) or 2 hr (ChIP assay) and subsequently stimulated with 50 ng/ml EGF for 30 min. When required, cells were pre-treated with MAP kinase inhibitor U0126 (10 µM) for 30 min before EGF stimulation.

### Chromatin Immunoprecipitation (ChIP) and ChIP-Chip

ChIP was performed essentially as described previously [26]. MCF-7 cells were grown to ∼80 to 90% confluence, cross-linked with 1% paraformaldehyde for 10 min at 37°C, and quenched in 125 mM glycine. Cell lysates were sonicated under conditions yielding fragments ranging from 200 bp to 800 bp. The material was clarified by centrifugation, diluted 10-fold in dilution buffer, and pre-cleared with protein A-agarose beads. The pre-cleared, chromatin-containing supernatant was used in immunoprecipitation reactions with antibodies against PADI4 (Sigma P4749), Elk-1 (Santa Cruz sc-22804), p-Elk-1 (Santa Cruz sc-135646), Ac-H4K5 (Upstate 07-327), normal rabbit IgG (Upstate 12-370) or without antibodies as a control. Ten percent of the supernatant was saved as reference control. Both the immunoprecipitated genomic DNA and the control DNA were cleared of protein and residual RNA by digestion with proteinase K and RNase (Roche), respectively. The DNA was then extracted with phenol∶chloroform∶isoamyl alcohol and precipitated with ethanol. For gene-specific ChIP analyses, quantitative real-time PCR (qPCR) was used to determine the enrichment of immunoprecipitated DNA relative to the input DNA using gene-specific primer sets to the specified regions. Each ChIP experiment was conducted a minimum of three times with independent chromatin isolates to ensure reproducibility. For the ChIP-chip analyses, ChIP was performed in MCF-7 cells stripped in MEM plus 5% charcoal-dextran-treated calf serum for 72 hrs. LM-PCR-amplified immunoprecipitated DNA and the reference DNA were hybridized to a human HG18 RefSeq promoter microarray from Nimblegen. The array contained all known well-characterized RefSeq gene promoters. The promoter region on this array was covered by 50–75 mer probes with a mean probe spacing of 100 bp. The detailed information is available on Nimblegen's website.

### Genomic Data Analyses

The genomic data analyses were performed using the statistical programming language R [47] essentially as described previously [21]. All data processing scripts are available upon request. The log_2_ ratio data from each array was subjected to lowess normalization [48]. The normalized data were scaled to equivalent sum of squares and then the between array mean log_2_ ratio was determined for each probe. An error model was generated using a 1 kb moving window with 250 bp steps in which both the mean probe log_2_ ratio and p-values were calculated for each window. The p-values were calculated using the nonparametric Wilcoxon signed-rank test. The TSS-anchored ChIP-chip heat maps were generated using 600 pb windows with 150 bp steps and were visualized (Figure 1B) with Java Treeview [49]. For genes with multiple TSSs, the most 5′ site was used. The raw and processed data from the ChIP-chip data described in this manuscript can be found on the GEO website (http://www.ncbi.nlm.nih.gov/geo/) using accession number GSE18755.

#### Identification of PADI4-bound and PADI4-unbound regions

Significant PADI4 peaks were defined as the center of three consecutive windows with positive means, at least six probes, the center window with a mean greater than either adjacent window, and all windows having p-values less than 0.016. Significant PADI4 troughs were defined as the center of three consecutive windows with negative means, at least six probes, the center window with a mean less than either adjacent window, and all windows having p-values less than 0.016. The use of these selection criteria were justified by a low FPR as determined by ChIP-qPCR (PADI4-peak FPR<15%; PADI4-trough FPR<15%).

#### Peak to TSS distances

The distance of PADI4 bound region to the closest TSS (Figure 1B) was determined using RefSeq gene annotations from the UCSC genome browser. A Fisher exact test was used to determine the significance of the patterns observed in the boundary histograms, comparing to the background pattern obtained by determining the distance of all windows with at least six probes to the closest TSS.

#### Expression-based classification of genes

For the expression-based categorization of genes, three replicates of previously described MCF-7 expression microarray data (Affymetrix U133A, GEO accession number GSE9253) were associated with the genes represented on the ChIP-chip array [26]. In cases where multiple probe sets corresponded to a given gene, all signals from the probe sets were averaged. For a gene on the ChIP-chip array to be marked as unambiguously expressed or unexpressed, all probe sets from all three expression array replicates corresponding to the gene must have been flagged unanimously present or absent, respectively. Any genes on the array not meeting these criteria were marked as ambiguous and were removed from the expression based categorization analysis.

#### Multiple ChIP-chip correlation analysis

Data series containing MCF-7 ChIP-chip data were downloaded from the Gene Expression Omnibus (GEO) website (http://www.ncbi.nlm.nih.gov/geo). The genomic data from the chosen series had to have been performed using a two-color platform (i.e. Nimblegen, Agelent). In total 362 ChIP-chip data sets from the following GEO series were included in the analysis: GSE1778, GSE2072, GSE6292, GSE7118, GSE8887, GSE2672, GSE4355, GSE4355, GSE10504, GSE3505, GSE5175, GSE6625, GSE8667, GSE12126, GSE6624, GSE6634, GSE8716, GSE12126, GSE5445, GSE9015, GSE12650, GSE5559, GSE8716, GSE6385, GSE8855, GSE9029, GSE2672, GSE5559, GSE13051. The data was processed with 1 kb windows identical to that of the PADI4 analysis. The spearman correlation between PADI4 and each of the downloaded ChIP-chip data sets was then determined. The score of each correlation was defined as the negative of the log_10_ of the p-value for the correlation.

#### Comparion of PADI4 ChIP-chip with Elk-1 ChIP-chip

In order to determine the enrichment of PADI4 at bona fide Elk-1 binding sites, Elk-1 peaks determined from Elk-1 ChIP-chip experiment were obtained from previously published sources [33]. The ChIP-chip data was filtered for regions of the genome covered by the PADI4 ChIP-chip array. An Elk-1 binding site was defined as co-occupied by PADI4 if it occurred within 1.5 kb of a PADI4 peak. A Fisher exact test was used to determine the significance of the enrichment of Elk-1binding sites near PADI4 peaks.

#### Enrichment of transcription factor binding sites (TFBS) analysis

In order to determine which TFBS were enriched near genes marked by PADI4 bound we used the conserved TFBS track in the UCSC Genome Browser (http://genome.ucsc.edu/cgi-bin/hgTables) [50], [51]. The training set for this analysis was composed of all DNA within 2 kb of a TSS that contained a PADI4 binding region. The background set was created by removing the training set from the DNA within 2 kb of all TSS represented on the ChIP-chip array. We used the functions of the Galaxy web site (http://main.g2.bx.psu.edu/root) to determine the instances of TFBS in both the background and training sets. The total number of bp marked by each specific TFBS in both the background and training sets was computed. This information was used to compute a p-value for the enrichment using the Fisher exact test. Sequence Logos for the 21 most enriched motifs presented in Figure 3A were generated by using the position weight matrices from the Transfac database (http://www.gene-regulation.com/pub/databases.html) and the R package, SeqLogo [52].

#### Functional classification and annotation of the candidate genes

The functional profiling of PADI4-assocaited transactive genes was performed using the DAVID Bioinformatics Resources 6.7 [53], [54]. 516 unambiguously expressed genes as having significant PADI4 peaks were uploaded into DAVID and the ‘functional annotation tool’ was used to identify enriched biological themes.

### Gene-Specific Expression Analyses

Total RNA was isolated from MCF-7 cells using Qiagen RNeasy Mini Kit in combination with on-column DNase treatment (ABI). High Capacity RNA-to-cDNA kit (ABI) was used to synthesize the first strand of cDNA. Quantitative real-time PCR (qPCR) was performed using Power SYBR Green PCR Master Mix (ABI) with gene-specific primers. All target gene transcripts were normalized to β-actin. *PADI4* transcription in PADI4-overexpressed MCF-7 cells was assessed using TaqMan Gene Expression Master Mix (ABI) with Taqman probes and primers (*PADI4*: Hs00202612_m1 and *GAPDH*: 4352934E, ABI). Each experiment was conducted three times with independent isolates of total RNA. The P-values were determined using a two-tailed paired Student's t-test. All of the primers used for the ChIP-qPCR and RT-qPCR assays were listed in Table S3.

### Small-Scale Chromatin Fractionation

PADI4-depleted and parallel control MCF-7 cells (90% confluence in 10 cm culture plates) were collected, washed with phosphate-buffered saline (PBS), and resuspended in 500 µl of buffer A [10 mM HEPES (pH 7.9), 10 mM KCl, 1.5 mM MgCl2, 0.34 M sucrose, 10% glycerol, 1 mM dithiothreitol, and protease inhibitor cocktail (Roche)]. Triton X-100 was added (0.1% final concentration), the cells were incubated on ice for 8 min, and nuclei were collected by centrifugation (5 min, 1,300× g, 4°C). The nuclei were washed once in buffer A and lysed for 30 min in buffer B (3 mM EDTA, 0.2 mM EGTA, 1 mM dithiothreitol, and protease inhibitor cocktail), and insoluble chromatin and soluble fractions were separated by centrifugation (5 min, 1,700× g, 4°C). The chromatin fraction was washed once with buffer B and resuspended in sodium dodecyl sulfate (SDS)-Laemmli buffer (and boiled for 10 min) for Western Blot analysis.

### Immunoprecipitation Assay

Flag-tagged PADI4 in pcDNA3.1 (+) or control vector were transfected into 293 cells using FuGENE 6. The whole cell lysates were immunoprecipitated with anti-Flag M2 affinity gel (Sigma A2220) or immunoprecipitated with anti-Elk-1 antibody. Immunoprecipitates were washed and analysed by western blot using anti-Elk-1, anti-phospho-Elk-1, and anti-PADI4 antibodies as indicated.

### Luciferase Reporter Assay

HEK293 cells were cotransfected using FuGENE 6 (Roche) with 0.05 µg of *c-Fos*-luciferase construct containing the SRE sequence as described previously [55], 0.25 µg of PADI4 (wild-type [WT] or enzymatically inactive mutant, mutation of Cys^645^ into Ser^645^, [CS]) in pcDNA3.1(+), and 0.25 µg of Elk-1 (wild-type [WT] or dominant-negative mutant, mutations of Ser^383^/Ser^389^ into Ala^383^/Ala^389^, [DN]) in pSG5 expression vectors [46]. For GAL4-driven luciferase assay, expression vector for the DNA-binding domain of GAL4 (CMV-GAL4) or GAL4-Elk-1 (full length) was transfected into HEK293 cells with 5xGAL4-driven luciferase reporter plasmid containing the minimal E1b promoter and plasmids for pcDNA3.1, PADI4 WT and C645S mutant. Lysates were assayed for luciferase activities 24 hours after transfection according to the supplier's protocol (Dual Luciferase, Promega) and normalized to protein concentration in the extract.

### PADI Assay and *In Vitro* ERK Kinase Assays

PADI4 proteins were expressed and purified from pET28b-PADI4 or pET28b-PADI4 (C645S) mutant using Ni-NTA Protein Purification System (Qiagen) according to manufacturer's instructions. The PADI assay was performed essentially as described previously [17]. The expressed Elk-1 was treated with PADI4 in PADI buffer containing 50 mM Tris.HCl, pH 7.6, 4 mM DTT, 4 mM CaCl_2_ at 37°C for 1 hr. The mutant PADI4 (CS) was used as the negative control. Coupled in vitro transcription/translations were performed from Elk-1 constructs using the TNT-coupled reticulocyte lysate system (Promega) with T7 RNA polymerase according to the instructions of the manufacturer. Both wild-type (WT) and dominant-negative (DN) Elk-1 cDNAs are in pSG5 expression vectors [46]. The *In vitro* ERK kinase assay was performed as described previously [55]. Briefly, after EGF treatment, JEG3 cells (The cell line is highly sensitive to EGF and has been shown to be good source for activated ERKs [56]) were washed in 1 ml of ice-cold PBS and lysed in radioimmune precipitation buffer containing 20 mM Tris (pH 8.0), 137 mM NaCl, 10% glycerol, 1% Nonidet P-40, 0.1% SDS, 0.5% deoxycholate, 2 mM EDTA, 5 mM sodium vanadate, 5 mM benzamidine, and 1 mM PMSF on ice for 10 min. The cell lysates were collected and cleared by centrifugation. ERK2 was immunoprecipitated by adding anti-ERK2 antibody (Santa Cruz sc-154) and 25 µl of protein A/G-agarose beads to clarified cell lysates. The beads were washed once in 1 ml of radioimmune precipitation buffer; twice in 1 ml of ice-cold Nonidet P-40 wash buffer; and once in 1 ml of kinase buffer containing 20 mM HEPES (pH 7.5), 20 mM MgCl_2_, 25 mM β-glycerol phosphate, 100 µM sodium vanadate, 20 µMATP, and 2 mM dithiothreitol. The reaction mixture (50 µl) contained the agarose beads suspended in kinase buffer, and substrate Elk-1 pretreated with PADI4. Samples were incubated for 30 min at 30°C with frequent mixing and the reaction was terminated with the addition of SDS loading buffer and boiled for 2 min, and analysed by western blot using anti-phospho-Elk-1 (p-Elk-1), anti-Elk-1, anti-phospho-ERK (p-ERK) (Cell Signalling E10), and anti-ERK2 antibodies as indicated.

## Supporting Information

Figure S1 PADI4 antibody specificity. (A) N-terminal amino acid alignment (1–15) for PADI family members. Conserved amino acids are denoted in red while amino acids conserved only between certain family members are shown in blue (http://bioinfo.genotoul.fr/multalin/multalin.html). (B) Western blot of MCF-7 cell extracts following transfection of either pcDNA3.1 (+) alone or pcDNA3.1 containing the flag-tagged PADI4 plasmid. The PADI4-Flag fusion protein was detected using either an anti-PADI4 antibody or an anti-Flag antibody. (C) MCF-7 cells were transfected with empty vector, pcDNA3.1 (+), or with vectors encoding different PADI family members. Immunoblots were performed using anti-PADI1 (Abcam ab24008), PADI2 (Proteintech 12110-1-AP), PADI3 (Abcam ab50246), and PADI4 (Sigma P4749) antibodies, respectively.
(DOC)

Figure S2 PADI4 is associated with known PADI4 bound gene promoters (*OKL38* and *p21*). ChIP-qPCR analysis of PADI4 binding at *OKL38* promoter, and two p53-binding sites on the *p21* gene promoter (PBS1 and PBS2) in MCF-7 cells.
(DOC)

Figure S3 Scatterplot showing the PADI4 ChIP-chip false positive rate for both PADI4 peaks and troughs (<15% with window P-value<0.016, respectively) as determined by ChIP-qPCR. The detection threshold for PADI4 peak identification is indicated by line.
(DOC)

Figure S4 Putative consensus sequence for each transcription factor. The top 21 motifs are listed in order of significance according to Figure 3A. Sequence logo images were created using the position weight matrices from the Transfac database (http://www.gene-regulation.com/pub/databases.html) and the R package, SeqLogo.
(DOC)

Figure S5 Western Blot with anti-Elk-1 antibody showing shRNA-mediated depletion of Elk-1 in HEK293 cells versus shRNA control knockdown cells. β-Actin was used as internal control showing the equal protein loading.
(DOC)

Figure S6 Dose-dependent effect of Cl-Amidine on *c-Fos* expression. Real-time RT-PCR analysis of *c-Fos* and *GAPDH* expression in serum-starved or EGF-stimulated MCF-7 cells with or without Cl-Amidine treatment.
(DOC)

Figure S7 EGF-induced *c-Fos* gene expression is inhibited by Cl-Amidine treatment in other breast cancer cells. Real-time RT-PCR analysis of *c-Fos* expression in serum-starved or EGF-stimulated BT474 and MCF10DCIS breast cancer cells with or without Cl-Amidine treatment.
(DOC)

Figure S8 Luciferase reporter assay with construct driven by the *c-Fos* promoter in HEK293 cells showing that PADI4 enzymatic activity facilitates Elk-1 mediated activation of *c-Fos*. Either wild type (WT) or C645S mutant (CS) PADI4 with N-terminal Flag tag and either wild type (WT) or dominant negative (DN) Elk-1 with N-terminal HA tag were co-transfected with the *c-Fos* reporter construct (*c-Fos* promoter fragment containing 531 nucleotides which begins 85 nucleotides downstream from the TATAA box and contains 446 nucleotides upstream of the TATAA box. SRE sequence was included in this construct). Empty vector was used to normalize the equal amount of plasmid DNA transfected. Luciferase activity was measured as described in Figure 4F. In the lower panel, the expressions of Flag-PADI4 and HA-Elk-1 fusion proteins were monitored by western lot using anti-Flag and anti-HA antibodies.
(DOC)

Table S1 PADI4 binding regions identified by ChIP-chip analysis (P-value<0.016). See separate Excel file.
(XLS)

Table S2 Gene ontology analysis for the transactive genes associated with PADI4 binding in MCF-7 cells. See separate Excel file.
(XLS)

Table S3 Details of PCR primers used for the ChIP-qPCR and RT-qPCR assays.
(DOC)
