# Supplementary material for: Genome-Wide Analysis Reveals PADI4 Cooperates with Elk-1 to Activate c-Fos Expression in Breast Cancer Cells
Source: PLoS Genet. 2011 Jun 2;7(6):e1002112. doi: 10.1371/journal.pgen.1002112 (PMC3107201; doi:10.1371/journal.pgen.1002112)
Supplement: Figure S4 — Putative consensus sequence for each transcription factor. The top 21 motifs are listed in order of significance according to Figure 3A. Sequence logo images were created using the position weight matrices from the Transfac database (http://www.gene-regulation.com/pub/databases.html) and the R package, SeqLogo. (DOC) [file pgen.1002112.s004.doc]

**Figure S4**

V$STAT1_01

V$ELK1_02

V$NFY_Q6

**
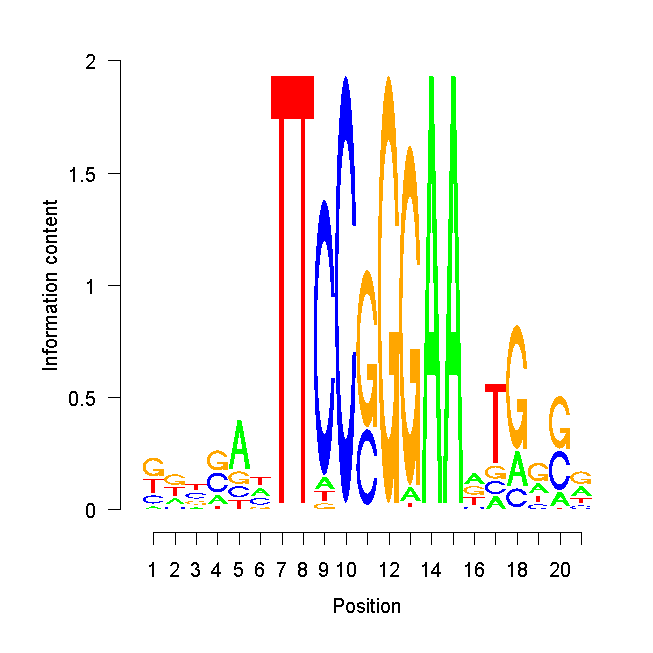

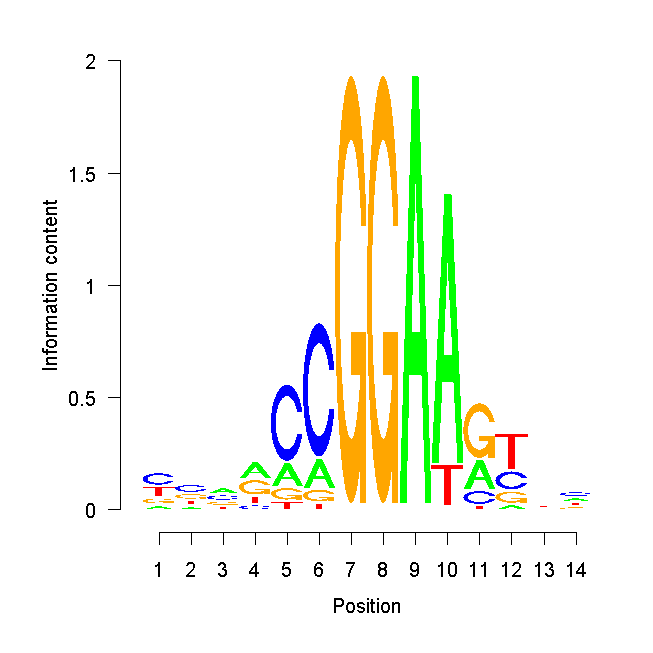

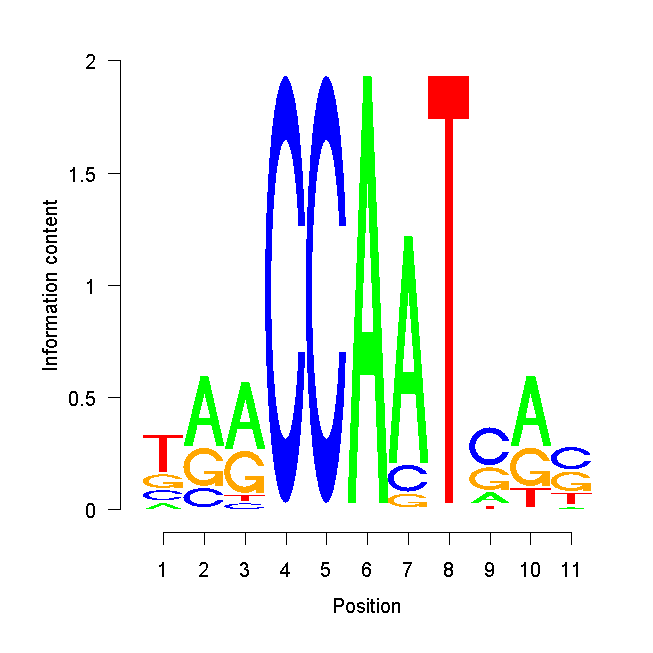
**

Information content

Information content

Information content

Position

0

0.5

1.0

1.5

2.0

0

0.5

1.0

1.5

2.0

0

0.5

1.0

1.5

2.0

Position

Position

V$CETS1P54_01

V$STAT3_01

V$PAX5_02


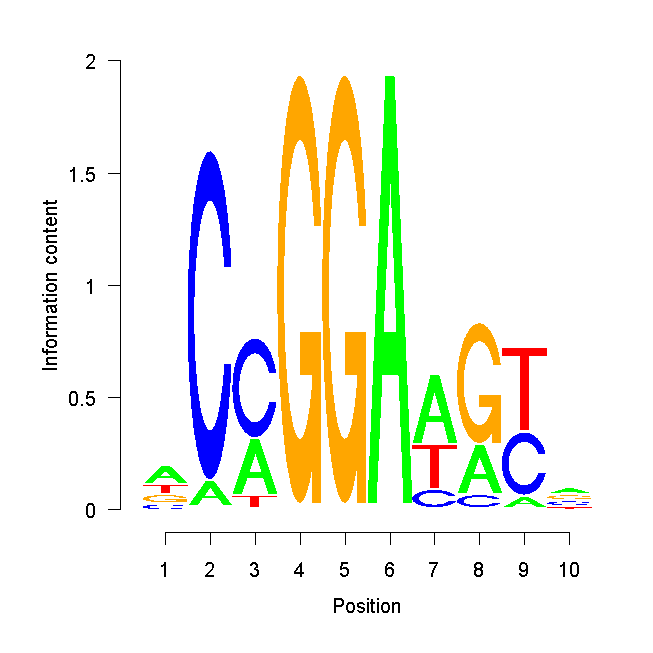

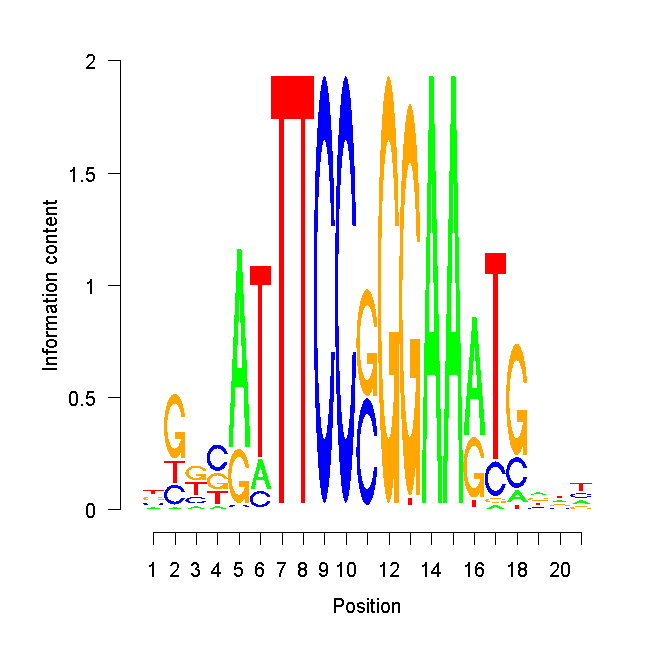

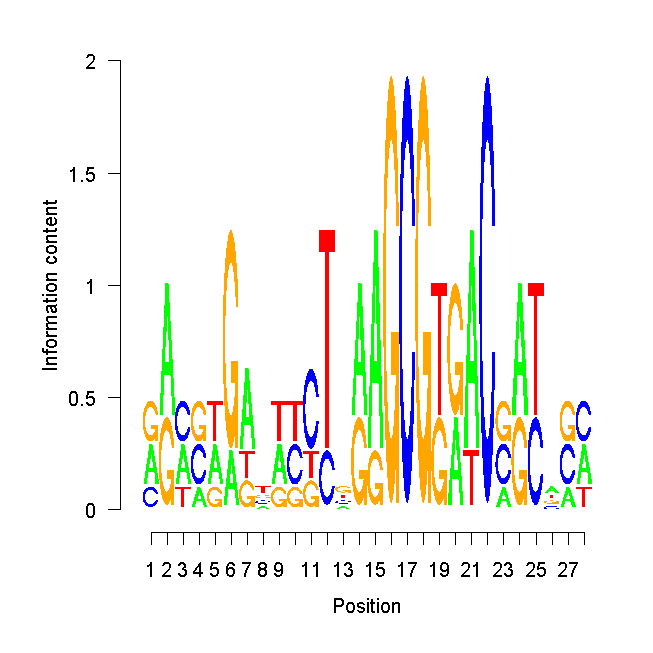


Information content

Information content

Information content

0

0.5

1.0

1.5

2.0

Position

Position

0

0.5

1.0

1.5

2.0

Position

0

0.5

1.0

1.5

2.0

V$AHRARNT_02

V$NRF2_01


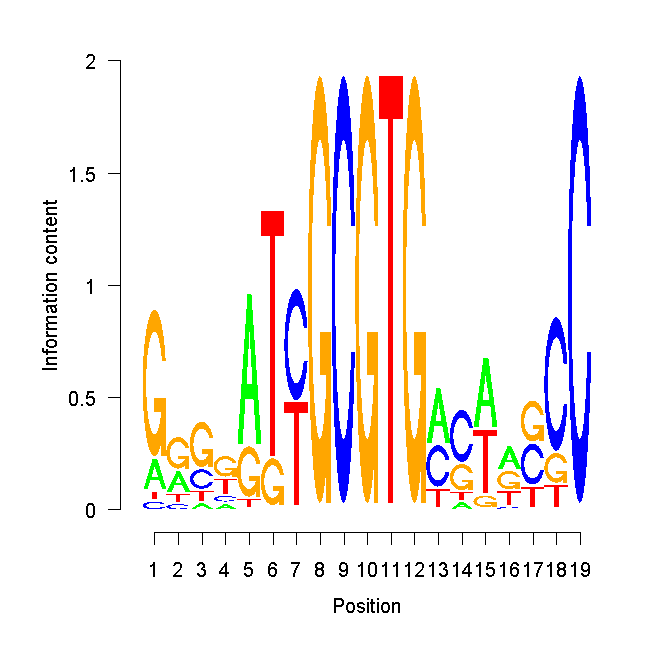

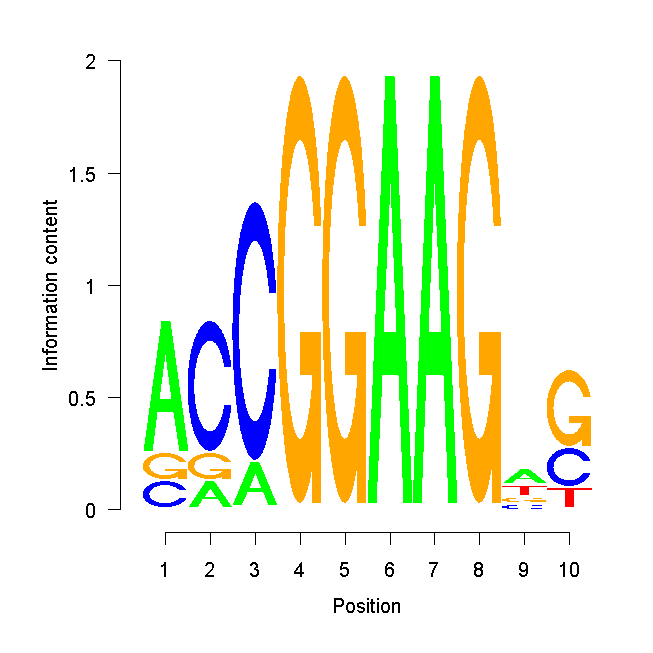

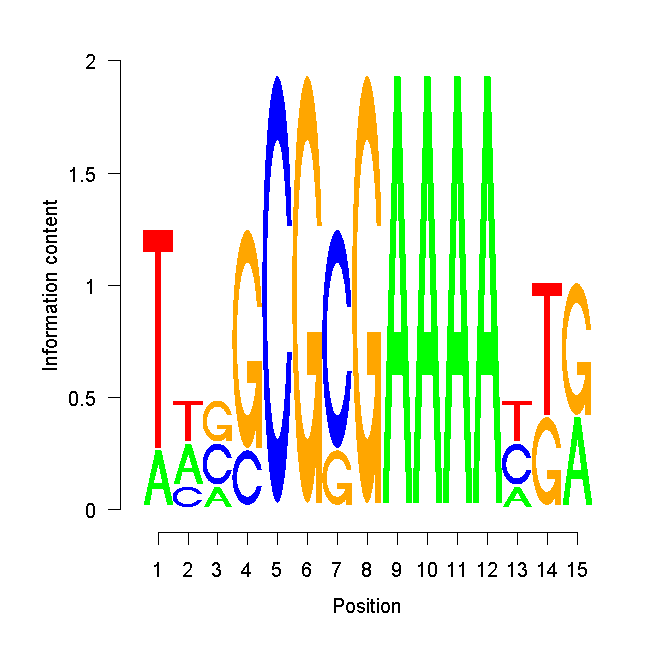


Information content

Information content

Information content

Position

Position

0

0.5

1.0

1.5

2.0

0

0.5

1.0

1.5

2.0

V$E2F_01

Position

0

0.5

1.0

1.5

2.0

V$YY1_01


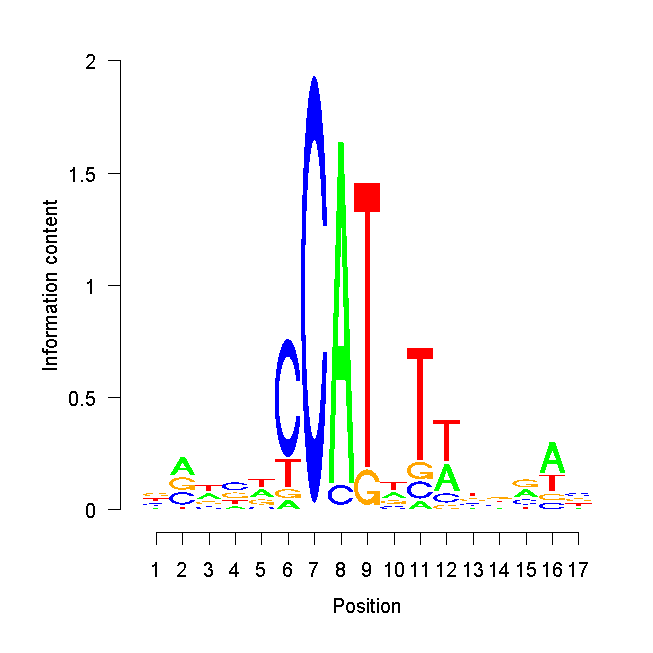

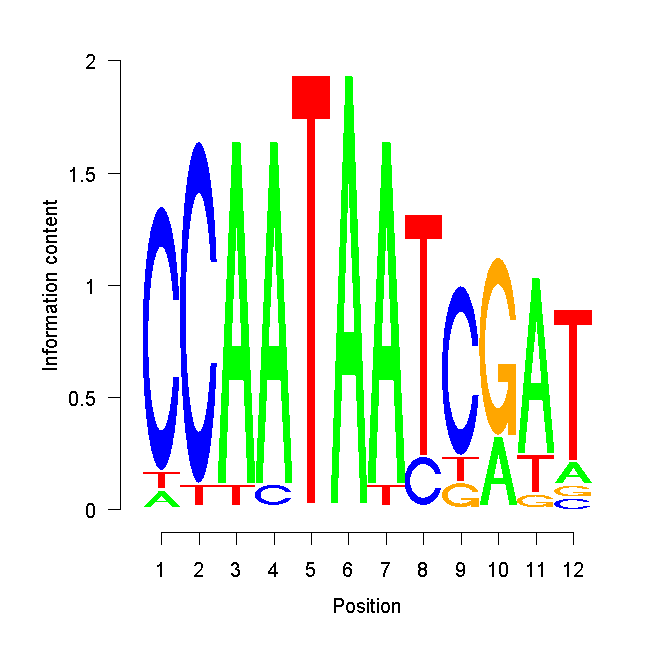

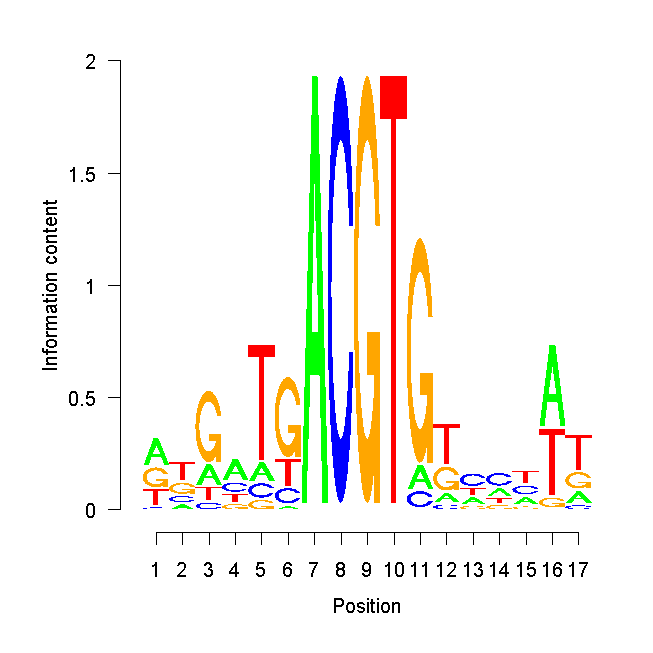


Information content

Information content

Information content

0

0.5

1.0

1.5

2.0

Position

V$CDP_01

Position

0

0.5

1.0

1.5

2.0

V$XBP1_01

Position

0

0.5

1.0

1.5

2.0

V$ZID_01


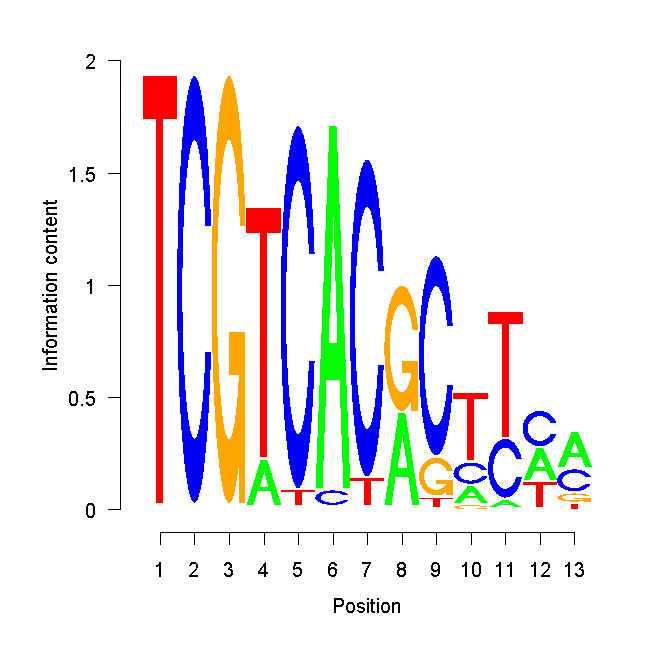

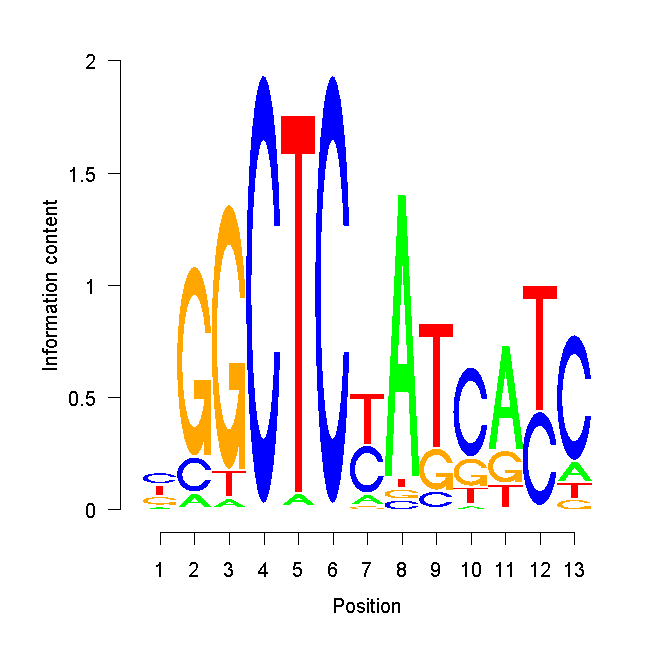

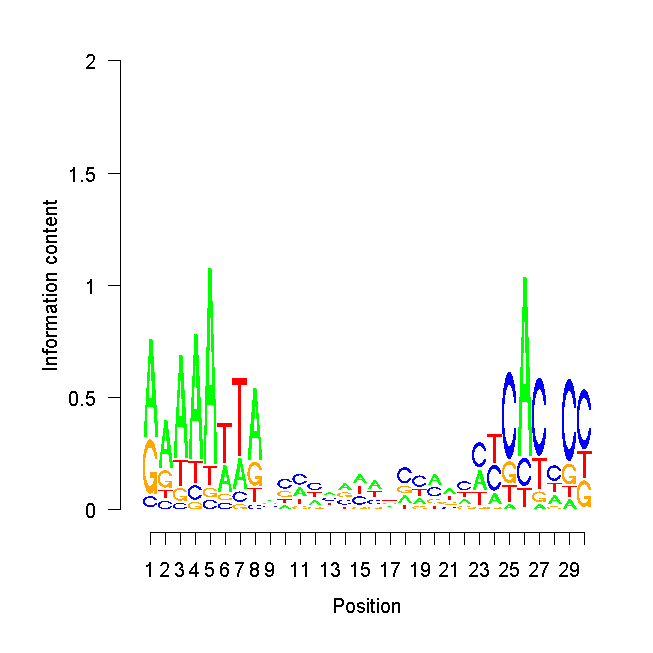


Information content

Information content

Information content

V$PAX3_01

Position

0

0.5

1.0

1.5

2.0

V$PAX4_04

0

0.5

1.0

1.5

2.0

0

0.5

1.0

1.5

2.0

Position

Position

V$CREB_Q4

V$TAXCREB_01

V$HLF_01


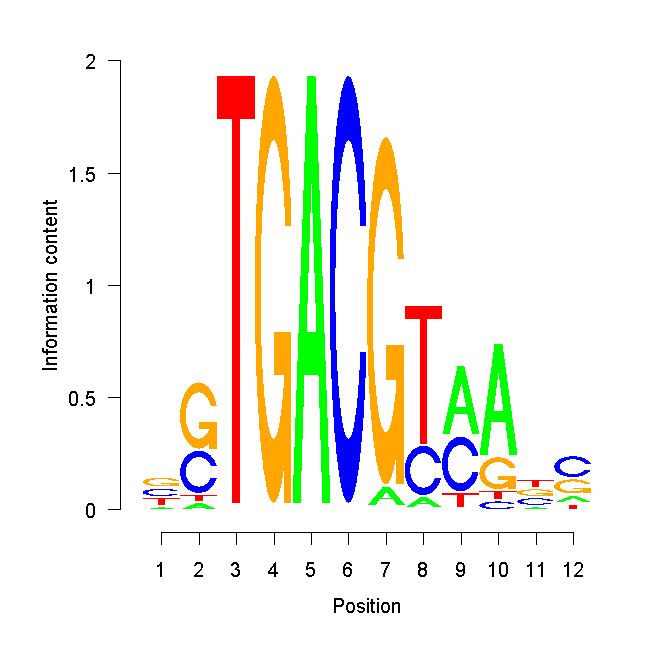

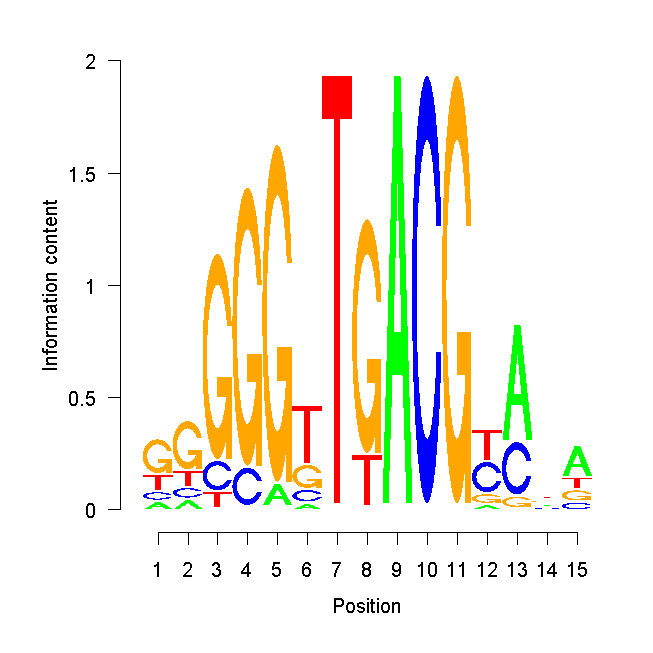

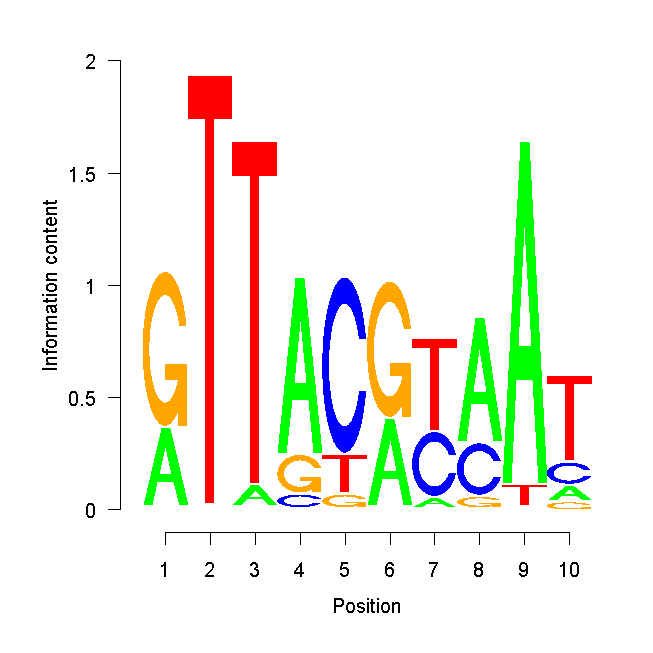


Information content

Information content

Position

Position

Position

0

0.5

1.0

1.5

2.0

0

0.5

1.0

1.5

2.0

0

0.5

1.0

1.5

2.0

V$CMYB_01

V$PPARG_01

V$CREBP1_Q2


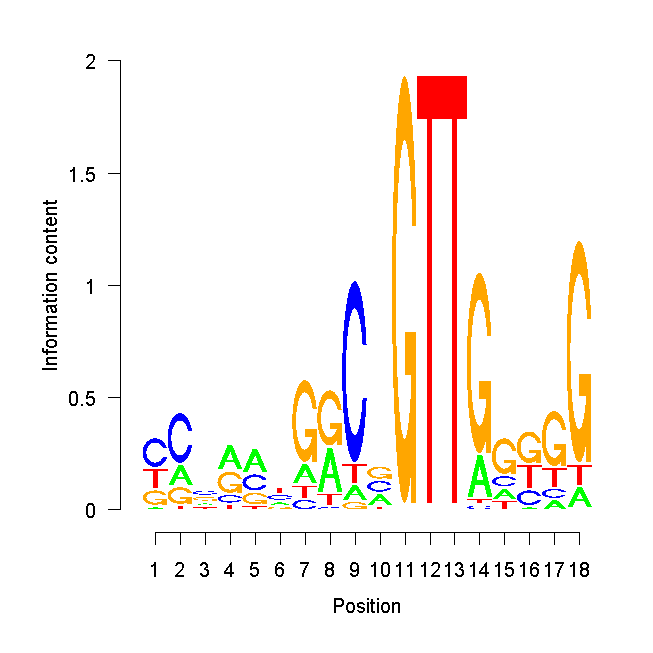

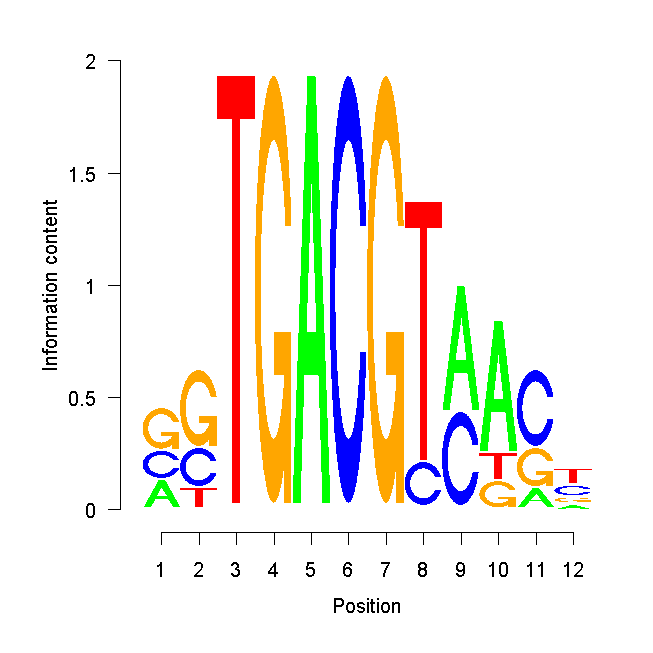

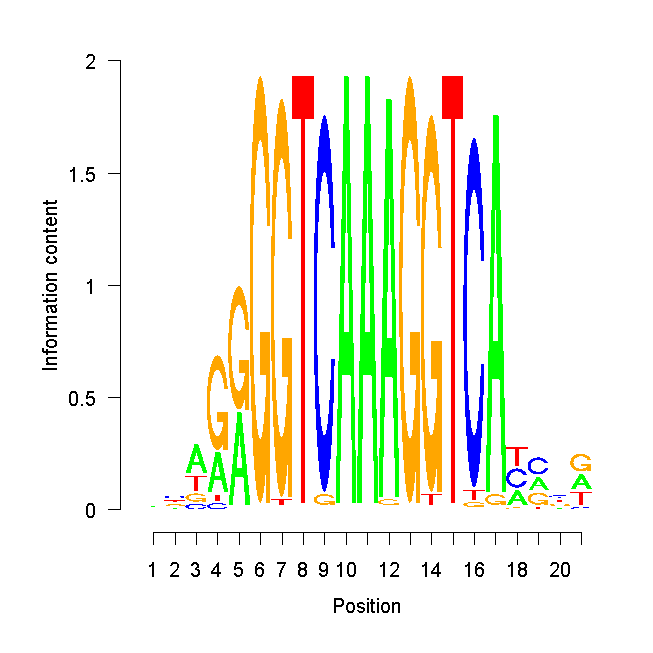


Information content

Information content

Information content

0

0.5

1.0

1.5

2.0

0

0.5

1.0

1.5

2.0

0

0.5

1.0

1.5

2.0

Position

Position

Position
