# Supplementary material for: Genome-Wide Analysis Reveals PADI4 Cooperates with Elk-1 to Activate c-Fos Expression in Breast Cancer Cells
Source: PLoS Genet. 2011 Jun 2;7(6):e1002112. doi: 10.1371/journal.pgen.1002112 (PMC3107201; doi:10.1371/journal.pgen.1002112)
Supplement: Figure S6 — Dose-dependent effect of Cl-Amidine on c-Fos expression. Real-time RT-PCR analysis of c-Fos and GAPDH expression in serum-starved or EGF-stimulated MCF-7 cells with or without Cl-Amidine treatment. (DOC) [file pgen.1002112.s006.doc]

**Figure S6**
