# Supplementary material for: Genome-Wide Analysis Reveals PADI4 Cooperates with Elk-1 to Activate c-Fos Expression in Breast Cancer Cells
Source: PLoS Genet. 2011 Jun 2;7(6):e1002112. doi: 10.1371/journal.pgen.1002112 (PMC3107201; doi:10.1371/journal.pgen.1002112)
Supplement: Figure S7 — EGF-induced c-Fos gene expression is inhibited by Cl-Amidine treatment in other breast cancer cells. Real-time RT-PCR analysis of c-Fos expression in serum-starved or EGF-stimulated BT474 and MCF10DCIS breast cancer cells with or without Cl-Amidine treatment. (DOC) [file pgen.1002112.s007.doc]

**Figure S7**
