# Supplementary material for: Genome-Wide Analysis Reveals PADI4 Cooperates with Elk-1 to Activate c-Fos Expression in Breast Cancer Cells
Source: PLoS Genet. 2011 Jun 2;7(6):e1002112. doi: 10.1371/journal.pgen.1002112 (PMC3107201; doi:10.1371/journal.pgen.1002112)
Supplement: Figure S8 — Luciferase reporter assay with construct driven by the c-Fos promoter in HEK293 cells showing that PADI4 enzymatic activity facilitates Elk-1 mediated activation of c-Fos. Either wild type (WT) or C645S mutant (CS) PADI4 with N-terminal Flag tag and either wild type (WT) or dominant negative (DN) Elk-1 with N-terminal HA tag were co-transfected with the c-Fos reporter construct (c-Fos promoter fragment containing 531 nucleotides which begins 85 nucleotides downstream from the TATAA box and contains 446 nucleotides upstream of the TATAA box. SRE sequence was included in this construct). Empty vector was used to normalize the equal amount of plasmid DNA transfected. Luciferase activity was measured as described in Figure 4F. In the lower panel, the expressions of Flag-PADI4 and HA-Elk-1 fusion proteins were monitored by western lot using anti-Flag and anti-HA antibodies. (DOC) [file pgen.1002112.s008.doc]

Figure S8
