# Supplementary material for: Genome-Wide Analysis Reveals PADI4 Cooperates with Elk-1 to Activate c-Fos Expression in Breast Cancer Cells
Source: PLoS Genet. 2011 Jun 2;7(6):e1002112. doi: 10.1371/journal.pgen.1002112 (PMC3107201; doi:10.1371/journal.pgen.1002112)
Supplement: Table S3 — Details of PCR primers used for the ChIP-qPCR and RT-qPCR assays. (DOC) [file pgen.1002112.s011.doc]

**Table S3. Details of PCR primers used for the ChIP-qPCR and RT-qPCR assays.**

**ChIP-qPCR primer list:**

| **Name** | **Primer Sequence** | **Positions of Amplicons** |
| --- | --- | --- |
| AHI1 TSSF | AATCCTGTGAGGAAGCAACG | Chr6:135861453+135861509 |
| AHI1 TSSR | AGCTCAGTTCGGTACGCATT |  |
| BAZ2A TSSF | CACCCCCATAACTCAGCATT | Chr12:55316814+55316878 |
| BAZ2A TSSR | TGCTTGTGCTGGTAGTTTGC |  |
| BLZF1 TSSF | CCCCCTGAAATACGGAGAAT | Chr1:167604172+167604223 |
| BLZF1 TSSR | TGCTTGTCCTCGGATTTACC |  |
| CCDC59 TSSF | AATACGTCACCAAGCCGAAG | Chr12:81276335+81276410 |
| CCDC59 TSSR | TAGCACAGGCAGGCTCAGTA |  |
| c-FOS TSSF | TTAGGACATCTGCGTCAGCA | Chr14:74814975+74815095 |
| c-FOS TSSR | AGCATTTCGCAGTTCCTGTC |  |
| c-FOS-A TSSF | CAGACACCCCCTTCAAATGT | Chr14:74814327+74814396 |
| c-FOS-A TSSR | AATCCCTGAGACACCAGCAG |  |
| c-FOS-B TSSF | GAGCAGTTCCCGTCAATCC | Chr14:74814930+74815094 |
| c-FOS-B TSSR | GCATTTCGCAGTTCCTGTCT |  |
| c-FOS-C TSSF | GCGAAATGCTCACGAGATTAG | Chr14:74815086+74815229 |
| c-FOS-C TSSR | GTAAACGTCACGGGCTCAA |  |
| c-FOS-D TSSF | GAGCCCGTGACGTTTACACT | chr14:74815213+74815294 |
| c-FOS-D TSSR | CAGATGCGGTTGGAGTACG |  |
| CSDE1 TSSF | AACATGGTGGCTGCATTACA | chr1:115102266+115102356 |
| CSDE1 TSSR | ACCCCTAGTGTCCAGGAGGA |  |
| CYP8B1 TSSF | TGACTGTATGCCCTTCCACA | chr3:42893188+42893250 |
| CYP8B1 TSSR | CCAGCATCAATGCCTCTTTT |  |
| DAPK3 TSSF | GACCCTCATCGATGGAACAC | chr19:3921462+3921539 |
| DAPK3 TSSR | CCGGTTCTTGGAAAATTCAG |  |
| EIF2B5 TSSF | TGCCTGCCACCTTCTCTATT | chr3:185335337+185335411 |
| EIF2B5 TSSR | GGAGATAGAACCGAGCCAGA |  |
| FBXO44 TSSF | TCCTCATATGCAGCCTCTCC | chr1:11635457+11635541 |
| FBXO44 TSSR | GGACTCACTGTGTGCTCTGG |  |
| FOXA1 TSSF | AAAAAGCCCCACTTTTGCTT | chr14:37134827+37134960 |
| FOXA1 TSSR | TCTTAGCCGCAGGTACGAGT |  |
| FTSJ1 TSSF | TACAGGGCAGGAGGCTAGTG | chrX:48220610+48220710 |
| FTSJ1 TSSR | GCTCCCCATTAAGCATTCAA |  |
| GAPDH TSSF | AAAAGCGGGGAGAAAGTAGG | chr12:6643512+6643672 |
| GAPDH TSSR | GCTGCGGGCTCAATT TATAG |  |
| HIPK1 TSSF | CAGCCCAATGTGAGGACTTT | chr1:114272495+114272567 |
| HIPK1 TSSR | CCCACAAGGCAGTTTTGTTT |  |
| HIST2H2AB TSS F | CAAAATGTCGCCCTGTTTTT | chr1:148126804+148126915 |
| HIST2H2AB TSS R | GGAGACAGGGAAACAGTCCA |  |
| HSPA8 TSSF | TGCCCTTACAAGACCCAATC | chr11:122438165+122438269 |
| HSPA8 TSSR | CTGCGGTGAGTGCGTTATC |  |
| JARID1A TSSF | TGTGGCTGTTTGTCTCCTTG | chr12:368846+368972 |
| JARID1A TSSR | GCTTCCCTCAATCCGTACCT |  |
| LARP7 TSS F | TCCCGAGAAACACTTCACCT | chr4:113777433+113777519 |
| LARP7 TSS R | GCGTTAATGACGTCACGTTG |  |
| OKL38 TSSF | CTTTTGCCTGCCCTTAACAG | chr16:83982622+83982731 |
| OKL38 TSSR | TCCCTGCCATCTTGTTTACC |  |
| PBS1-TSSF | AGCAGGCTGTGGCTCTGATT | chr6:36644189+36644279 |
| PBS1-TSSR | CAAAATAGCCACCAGCCTCTTCT |  |
| PBS2-TSSF | CTGTCCTCCCCGAGGTCA | chr6:36645078+36645164 |
| PBS2-TSSR | ACATCTCAGGCTGCTCAGAGTCT |  |
| PEF1 TSSF | CTGTCAATCTTGGGTGCTGT | chr1:31883159+31883261 |
| PEF1 TSSR | CGGAAGCTCCATCAATCACT |  |
| PNN TSSF | AGATGACTGCCTGGCTGACT | chr14:38714031+38714084 |
| PNN TSSR | AACCTGCAGCCTTTGTGACT |  |
| PRCC-A TSSF | TCACCTCGGAATTGTTAGGC | chr1:155003716+155003827 |
| PRCC-A TSSR | GTCCGAGGAAGGATTTAGGG |  |
| PRCC-B TSSF | CTTTCGTTCCCGACATCACT | chr1:155003567+155003636 |
| PRCC-B TSSR | CCCCTTGGCCACTAAGATTT |  |
| PRDX1 TSSF | CGGGTGCGGAAAAATACTAA | chr1:45760302+45760356 |
| PRDX1 TSSR | ACTCCAACCTCAGCCATCC |  |
| RPL30 TSSF | GTAGGAGCCCACTCACCAAC | chr8:99126896+99126978 |
| RPL30 TSSR | TGAAGAGCTTTGCATTGTGG |  |
| SEPN1 TSSF | TCTAGAGCCACCCCATGGTA | chr1:25998343+25998452 |
| SEPN1 TSSR | TGGGTGGTGTTGAGGACATA |  |
| SORCS3 TSSF | GCCTGCCTAGTTCTCAGCAT | chr10:106389990+106390051 |
| SORCS3 TSSR | TGTGTTTGCATTTCCAGGAG |  |
| TGFB1I1 TSSF | CCACTTTGTGGCTGGAGACT | chr16:31390153+31390270 |
| TGFB1I1 TSSR | CAAGACAAAGCCCTTCCTCA |  |
| TRIM37 TSSF | ACTTGCGTTCCACCTACCAA | chr17:54539483+54539565 |
| TRIM37 TSSR | CGTCCTCTCACCACCGTAAT |  |
| WSB2 TSSF | TGGGGAGGACGTAAGAATTG | chr12:116984076+116984159 |
| WSB2 TSSR | CATTACCGGAGCCATTGAGT |  |
| XKR4 TSSF | GTCATCCTCTCCCTCGGAGT | chr8:56177139+56177224 |
| XKR4 TSSR | ACCTGCACATTCCTCTCCTC |  |
| ZNF141 TSSF | CCCAAGCAGACAAGTGTCAA | chr4:320020+320078 |
| ZNF141 TSSR | GTGTATGCTCACGCAGTGCT |  |
| ZNF566 TSSF | AGTTGCTGGTAAAGCCCTGA | chr19:41672267+41672330 |
| ZNF566 TSSR | TTCTCGCGGTAATTCAGCTT |  |

RT-qPCR primer list:

| **Gene** | **Forward Primer** | Reverse Primer |
| --- | --- | --- |
| *ACTB* | ccaaccgcgagaagatga | ccagaggcgtacagggatag |
| *c-FOS* | ggggcaaggtggaacagt | tctccgcttggagtgtatca |
| *CSDE1* | agcccaaataaaggcaaagag | ccccacagtcatcataagca |
| *FBXO44* | cctctggggaccttccag | tggagaatgtgtgggagacc |
| *FOXA1* | agggctggatggttgtattg | accgggacggaggagtag |
| *GAPDH* | agccacatcgctcagacac | gcccaatacgaccaaatcc |
| *HSPA8* | tttttgtggcttccttcgtt | tcccttggacatggttgc |
| *PEF1* | tcgggctccattagctacac | cagagcgtgggcagtagc |
| *PNN* | aatccactgttgctactgaaagg | tcattttcaacctgctttctctc |
| *PRDX1* | cactgacaaacatggggaagt | tttgctcttttggacatcagg |
| *RPL30* | aacaactgcccagctttga | atgctgtgcccagttcaata |
| *SORCS3* | agaggaggaaaggcaatgaag | ttggttgagagcattaaacagtg |
